# Altered Gene Expression of the Parasitoid *Pteromalus puparum* after Entomopathogenic Fungus *Beauveria bassiana* Infection

**DOI:** 10.3390/ijms242317030

**Published:** 2023-12-01

**Authors:** Lei Yang, Jinting Li, Lei Yang, Xiaofu Wang, Shan Xiao, Shijiao Xiong, Xiaoli Xu, Junfeng Xu, Gongyin Ye

**Affiliations:** 1 State Key Laboratory for Managing Biotic and Chemical Threats to the Quality and Safety of Agro-Products, Key Laboratory of Traceability for Agricultural Genetically Modified Organisms, Ministry of Agriculture and Rural Affairs, Zhejiang Academy of Agricultural Sciences, Hangzhou 310021, China; 2 State Key Laboratory of Rice Biology and Breeding & Ministry of Agricultural and Rural Affairs Key Laboratory of Molecular Biology of Crop Pathogens and Insects, Institute of Insect Sciences, Zhejiang University, Hangzhou 310058, China

**Keywords:** fungi, infection, survival rate, host responses, transcriptome analysis

## Abstract

Both parasitoids and entomopathogenic fungi are becoming increasingly crucial for managing pest populations. Therefore, it is essential to carefully consider the potential impact of entomopathogenic fungi on parasitoids due to their widespread pathogenicity and the possible overlap between these biological control tools during field applications. However, despite their importance, little research has been conducted on the pathogenicity of entomopathogenic fungi on parasitoids. In our study, we aimed to address this knowledge gap by investigating the interaction between the well-known entomopathogenic fungus *Beauveria bassiana*, and the pupal endoparasitoid *Pteromalus puparum*. Our results demonstrated that the presence of *B. bassiana* significantly affected the survival rates of *P. puparum* under laboratory conditions. The pathogenicity of *B. bassiana* on *P. puparum* was dose- and time-dependent, as determined via through surface spraying or oral ingestion. RNA-Seq analysis revealed that the immune system plays a primary and crucial role in defending against *B. bassiana*. Notably, several upregulated differentially expressed genes (DEGs) involved in the Toll and IMD pathways, which are key components of the insect immune system, and antimicrobial peptides were rapidly induced during both the early and late stages of infection. In contrast, a majority of genes involved in the activation of prophenoloxidase and antioxidant mechanisms were downregulated. Additionally, we identified downregulated DEGs related to cuticle formation, olfactory mechanisms, and detoxification processes. In summary, our study provides valuable insights into the interactions between *P. puparum* and *B. bassiana*, shedding light on the changes in gene expression during fungal infection. These findings have significant implications for the development of more effective and sustainable strategies for pest management in agriculture.

## 1. Introduction

In recent years, entomopathogenic fungi have emerged as safe and environmentally friendly alternatives to chemical agents in pest control [1,2]. They are important components of integrated pest management (IPM) programs that aim to effectively and sustainably manage pest populations [3]. Additionally, parasitoids are considered vital tools in IPM programs as they parasitize and kill their insect hosts, reducing the host population and promoting a more balanced ecosystem. There is a growing interest in using parasitoids in combination with other IPM methods, including the application of entomopathogenic fungi in agricultural fields [4]. Therefore, it is crucial to identify important gene clusters involved in fungal defense and to carefully consider the potential effects of entomopathogenic fungi on parasitoids to ensure compatibility and maximize efficacy in insect biocontrol.

Fungi infest insects through one of three invasive pathways, the epidermal, digestive, or respiratory pathway, with direct invasion through the epidermis being the primary method of infestation [5,6]. Among these fungi, *Beauveria bassiana*, a well-known bio-insecticide, stands out for its ability to parasitize over 700 insect species from 149 families, owing to its broad host range [7]. *Beauveria bassiana* utilizes sophisticated strategies to overcome host defenses [8]. Accordingly, under long-term selection, insects have developed various approaches to resist fungal infections. The cuticular integument, mainly composed of chitin microfibrils and structural cuticular proteins, serves as an effective barrier against pathogens [9]. Fungi strongly affect the expression levels of genes related to cuticle formation in insects [10]. Additionally, the insect cuticle surface contains several defensive compounds, including toxic lipids, phenols and enzyme inhibitors, interspersed within the cuticle matrix, which fungi must cope with for successful virulence [11].

The immune system plays a major role in repelling fungal infections. Upon fungal invasion, insect cuticle immune defenses are initiated, leading to the subsequent activation of enzymes and the synthesis of melanin [12,13]. When the first line of defense is breached, systemic cellular and humoral immune defense functions come into play. The Toll, IMD, and JAK/STAT pathways are core immune signaling pathways with several key regulators in these immune pathways, having demonstrated their essential roles in inhibiting fungal proliferation through RNA knockdown assays [14,15]. Furthermore, the induction of antimicrobial peptides (AMPs) and the activation of prophenoloxidase and heat shock proteins (HSPs) have also been observed as effective defense mechanisms against invading microbes in insects [16,17].

In addition, hemolymph coagulation, apoptosis, and autophagy have also emerged as central processes in the clearance of *B. bassiana* from hosts [18,19]. Moreover, various antioxidant enzymes, including SODs, catalases (CATs), and peroxidases (HPXs), function against stress responses and protect the insect’s body from harsh environmental impacts. Previous studies have shown a potential interplay between stress and immune responses, possibly mediated by certain signal transduction pathways [20].

Additionally, besides the physical barriers and innate immune systems mentioned above, a multitude of gene families participate in various other biological systems in infected insects. However, the mechanisms by which these genes contribute to the responses against fungal invasion remain largely unclear. Therefore, a comprehensive study is required to unveil the underlying mechanisms that go beyond basic immunity.

Fungi produce complex toxins to overcome the defensive barriers of insects. In response, insect hosts have developed detoxification mechanisms that involve modifying the chemical and physical properties of fungal toxins to reduce their toxicity to the insects [21]. This detoxification process involves members of conserved superfamilies such as cytochrome P450s (P450s), glutathione S-transferases (GSTs), carboxylesterase (CarEs), and ATP-binding cassette (ABC) transporters, which work synergistically as detoxification tools [22]. Furthermore, several insect species have developed strategies to recognize and respond to volatiles from *B. bassiana* [23,24]. Olfaction has been associated with the perception of fungal volatiles. Chemosensory proteins (CSPs) and odorant-binding proteins (OBPs) are ligand carrier proteins that transport odorants to receptors and modulate the insect’s behavioral responses [25]. Olfactory proteins have shown increased abundance in migratory locusts infected with *Metarhizium anisopliae* and are candidates for mediating pathogen detection [26]. These data underscore the importance of olfactory proteins in mediating insect immune reactions.

Although most published studies have considered the detrimental effects of *B. bassiana* on parasitoids under laboratory conditions, a small amount of research suggests that *B. bassiana* does not significantly impact the growth and development of parasitoids [27,28,29]. Moreover, limited studies have focused on the altered gene expression profiles in fungus-infected parasitoids, and there is currently a lack of information on the underlying pathways involved in insect-fungi interactions. In this study, we focused on an endoparasitoid wasp named *Pteromalus puparum*, which is known for its ability to parasitize a wide range of Lepidoptera [30]. Before considering the combination between *P. puparum* and fungi, it is crucial to evaluate their interactions. With the current lack of information on the impact of *B. bassiana* on *P. puparum*, we conducted an experiment to assess the influence of *B. bassiana* on the adult stage of these wasps. Additionally, RNA-Seq has emerged as a powerful tool for high-throughput gene expression analysis, providing valuable insights into the genes involved in resisting fungal infections. Previous research has revealed a comprehensive genome of *P. puparum* at the chromosomal level [31], and identified several genes involved in immunity, cuticle formation, and detoxication, paving the way for a deeper molecular analysis of this parasitoid [32,33]. By integrating genomic and transcriptomic data, we can gain a comprehensive view of the underlying interaction mechanisms between *P. puparum* and the fungus. Our study aimed to investigate the pathogenicity of *B. bassiana* on *P. puparum*. Illumina sequencing revealed a vast array of differentially expressed genes (DEGs) between infected and uninfected parasitoid adults. Gene ontology (GO) and KEGG enrichment analysis were performed, and mechanisms highly related to fungal infection were analyzed. This study contributes to our understanding of *P. puparum* to fungal pathogen infection and provides insights that elucidate the intricate molecular mechanisms underpinning the interactions between *P. puparum* and *B. bassiana*.

## 2. Results

### 2.1. Parasitoid Survival Post-Exposure to Fungal Conidia

**Figure 1:**
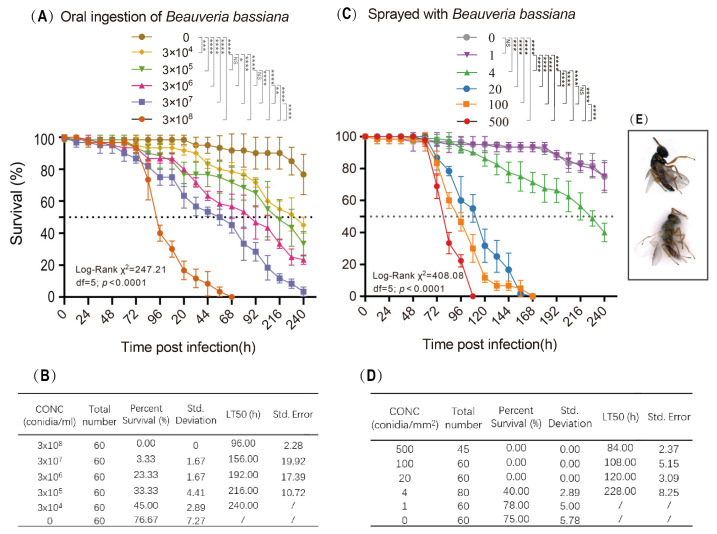
Virulence of the entomopathogenic fungus *Beauveria bassiana* against the parasitoid wasp *Pteromalus puparum*. (**A**,**C**) represent the survival rates of *P. puparum* exposed to different doses of *B. bassiana* via the oral-take method and the spraying method, respectively. The survival rates depict the percentage of surviving wasps over time after exposure to different concentrations of *B. bassiana* conidia. The log rank test was used to assess differences in survival rates between different treatments. Significant differences were represented by a definite *p* value and asterisks (* *p* < 0.05; ** *p* < 0.01; *** *p* < 0.001; **** *p* < 0.0001; NS, not significant, *p* > 0.05). Error bars represent the means ± standard deviations from three biological replicates. (**B**,**D**) illustrate the LT50 (median lethal time) for each treatment group. The LT50 represents the time it takes for 50% of the exposed wasps to die after infection with *B. bassiana*. (**E**) A comparison of the symptoms of dead *P. puparum* that were unexposed to fungi (**above**) and those exposed to *B. bassiana* (**below**). The symptoms observed in the dead wasps exposed to the fungi indicate signs of fungal infection.

Experiments were conducted to investigate the impact of the fungus *B. bassiana* on the parasitoid wasp *P. puparum* through two routes of exposure: the oral intake of sucrose water containing fungal conidia and surface spraying with a conidial suspension. In the oral ingestion experiment, we measured conidia concentration in conidia/mL, while in the spraying experiments, we quantified it as conidia/mm^2^, which allowed us to precisely account for the number of fungal spores that effectively adhered to the insects. The survival rates of all *B. bassiana*-treated parasitoids showed significant differences from those of the control groups, except for those exposed to a dose of one conidia/mm^2^ *B. bassiana* via spraying (Figure 1). The effect of *B. bassiana* on the lifespan of *P. puparum* was found to be dose-dependent, with dead wasps exhibiting fungal outgrowths covering their surfaces four days after death (Figure 1E).

In the feeding method, significant differences in the survival rates of *P. puparum* were observed among all six groups (Log-Rank χ^2^ = 247.21, df = 5, *p* < 0.0001) (Figure 1A). Pairwise multiple comparison analysis highlighted significant differences between most groups, with the exceptions of the groups infected with 3 × 10^4^ and 3 × 10^5^ conidia/mL *B. bassiana*, as well as the groups infected with 3 × 10^5^ and 3 × 10^6^ conidia/mL *B. bassiana*. The LT50 decreased as the doses of fungal conidia increased. The LT50 of *P. puparum* infected with 3 × 10^8^ conidia/mL *B. bassiana* was only 96 h, whereas the LT50 for those infected with 3 × 10^4^ conidia/mL *B. bassiana* was 240 h. Similarly, there were statistically significant differences in the survival rates of *P. puparum* exposed to different doses of *B. bassiana* administered via spraying (Log-Rank χ^2^ = 408.08, df = 5, *p* < 0.0001) (Figure 1B). Pairwise multiple comparison analysis revealed that the survival rates of *P. puparum* sprayed with 100 conidia/mm^2^ *B. bassiana* did not significantly differ from those sprayed with 20 conidia/mm^2^ *B. bassiana*. The LT50 of infected *P. puparum*, infected through the spraying method, exhibited a negative correlation with the doses of *B. bassiana*.

The results from both methods (feeding and spraying) demonstrated a dose-dependent impact of *B. bassiana* on the lifespan of *P. puparum*, regardless of the mode of application of the fungal conidia. The spraying method was deemed more accurate for infecting adult parasitoids due to its better timing accuracy and the uniformity of infection. Consequently, subsequent experiments focused on samples treated with the spraying method.

### 2.2. Overview of Sequencing Data

**Figure 2:**
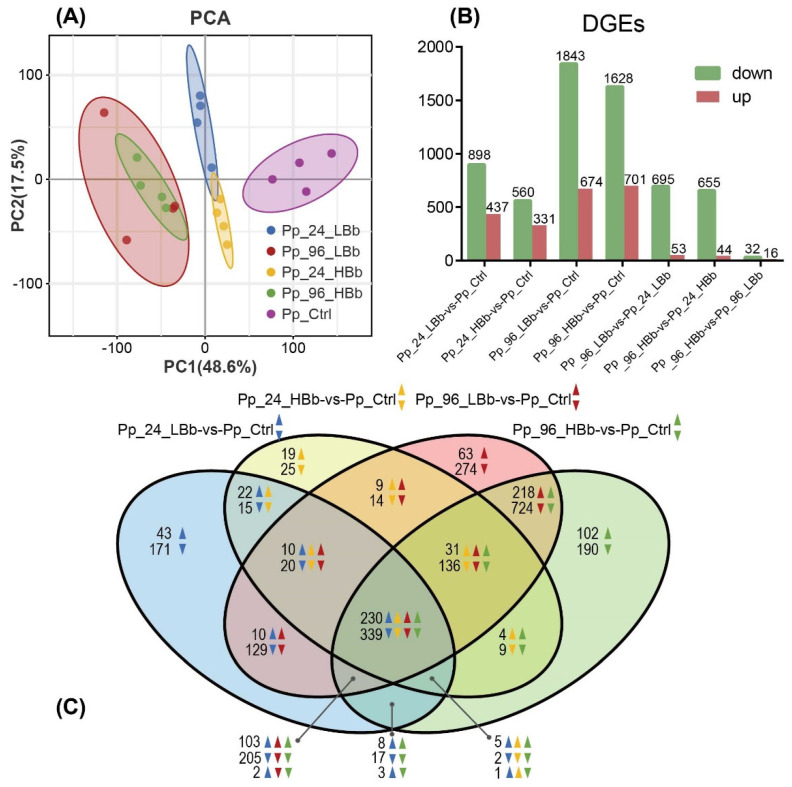
Summary of RNA-Seq analyses. The experiments involved *Pteromalus puparum* with high-concentration *Beauveria bassiana* (HBb) and low-concentration *B. bassiana* (LBb) and sampling at two different time points: 24 h and 96 h post-infection. Uninfected *P. puparum* served as controls for comparison. (**A**) Principal component analysis from control and *Bb*-infected parasitoids. (**B**) Numbers of the DEGs. The number on the column indicates the total up- and downregulated DEGs. (**C**) Venn diagram shows the distribution of DEGs among *P. puparum* infected with high- and low-concentration *B. bassiana* at early and late infection stages.

Transcriptome analysis using RNA-Seq generated a substantial number of clean reads per library, ranging from 40 to 56 million reads. The data exhibited high quality, with Q20 values exceeding 96.4% (Table S1). The clean data were mapped to both the reference genome of *P. puparum* and *B. bassiana*. A significant proportion, specifically 92.85%, was successfully mapped to the *P. puparum* genome (Table S1). However, the mapping rate to the *B. bassiana* genome was relatively low, ranging from 0.03% to 0.07% in Pp_96_LBb samples and from 0.1% to 0.23% in Pp_96_HBb samples (Table S1). Principal coordinate analysis (PCA) was conducted, showing that the first and second principal coordinates accounted for 48.6% and 17.5% of the data variance, respectively (Figure 2A). The PCA clearly differentiated between the infected and non-infected parasitoid samples and further separated the infected samples based on the time course of the infection.

### 2.3. Analysis of DEGs

In the RNA-Seq analysis, in total, 1335 genes, 2517 genes, 891 genes, and 2329 genes were identified as differentially expressed in Pp_24_LBb, Pp_96_LBb, Pp_24_HBb, and Pp_96_HBb groups, respectively, compared to Pp_Ctrl groups (Figure 2B). Notably, the majority of the DEGs exhibited downregulation in response to fungal infection, indicating a suppression of gene expression in the infected parasitoids. This suggests that *B. bassiana* infection has a significant impact on the gene expression profile of *P. puparum*.

The Venn diagram analysis revealed that 569 DEGs were shared among all treated samples. Among these shared DEGs, 230 genes were upregulated in all samples, while 339 genes were downregulated in all samples (Figure 2C). The significant overlap of DEGs suggests common transcriptional responses to *B. bassiana* infection across different conidial concentrations and time points. Furthermore, the examination of correlation coefficients for the log_2_ fold changes in the expression levels between each pair of samples showed strong and positive correlations, indicating a consistent gene expression response across the different treated samples (Table S2). This consistency suggests a robust and coordinated transcriptional response of *P. puparum* to *B. bassiana* infection.

### 2.4. GO and KEGG Enrichment Analysis

**Figure 3:**
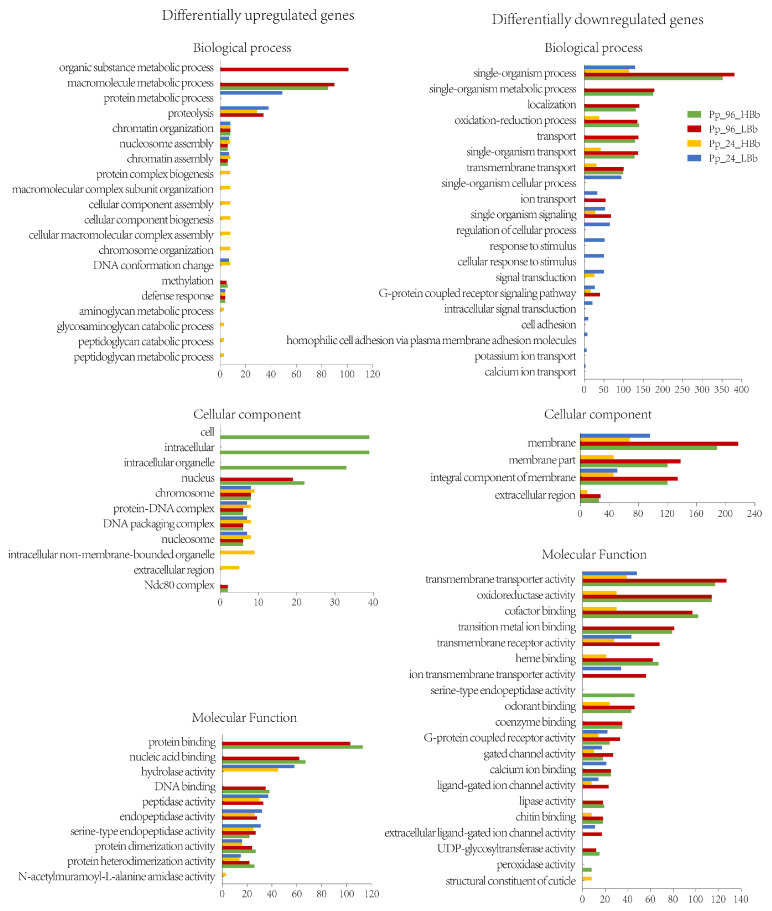
GO enrichment analysis of the upregulated and downregulated DEGs from HBb- and LBb-treated *Pteromalus puparum* both 24 h and 96 h post-infection. *p*-value < 0.05 was used as the threshold of significant difference.

The GO enrichment analysis provided insights into the potential biological functions of the DEGs in response to *B. bassiana* infection. Among the DEGs, 340 upregulated genes and 901 downregulated genes were enriched in at least one challenged sample with a significance cutoff of *p* < 0.05 (Tables S3 and S4). The top 20 enriched GO terms in each category were selected to draw the enrichment bar plot (Figure 3). Commonly upregulated enriched GO terms in all infected sample included chromatin organization, nucleosome assembly, chromatin assembly, defense response, serine-type endopeptidase activity, and protein dimerization activity. Conversely, commonly downregulated GO terms across infected samples were single-organism process, membrane, transmembrane transporter activity, G-protein coupled receptor activity, and gated channel activity.

The GO enrichment analysis not only identified common enriched GO terms across different *B. bassiana* infection conditions but also revealed sample-specific enriched GO terms. Some of the specific upregulated enriched GO terms include cellular response to stimulus and cell adhesion in the Pp_24_LBb sample, the structural constituent of the cuticle in the Pp_24_HBb sample, and peroxidase activity in the Pp_96_HBb sample. These specific terms indicate unique aspects of the host response to fungal infection at different time points and concentrations. Overall, the GO enrichment analysis provided insights into both common and specific biological processes, cellular components, and molecular functions that were differentially regulated upon *B. bassiana* infection under various conditions.

The KEGG pathway analysis revealed 303 DEGs that were significantly enriched in 32 pathways, with a *p*-value of <0.05. Interestingly, the pathway that included the largest number of DEGs was found to be related to human cancer. Additionally, several other prominent pathways were identified, including those related to signal transduction, substance dependence, the nervous system, and the immune system (Figure S1). These findings suggest that *B. bassiana* infection can have a wide-ranging impact on the molecular pathways and processes within *P. puparum*, some of which may be analogous to those involved in human cancer and immune responses. Further investigations of these pathways could provide valuable insights into the host-parasite interaction dynamics and the parasitoid’s defense mechanisms against fungal infection.

### 2.5. Differentially Expressed Immunity-Related Genes

**Figure 4:**
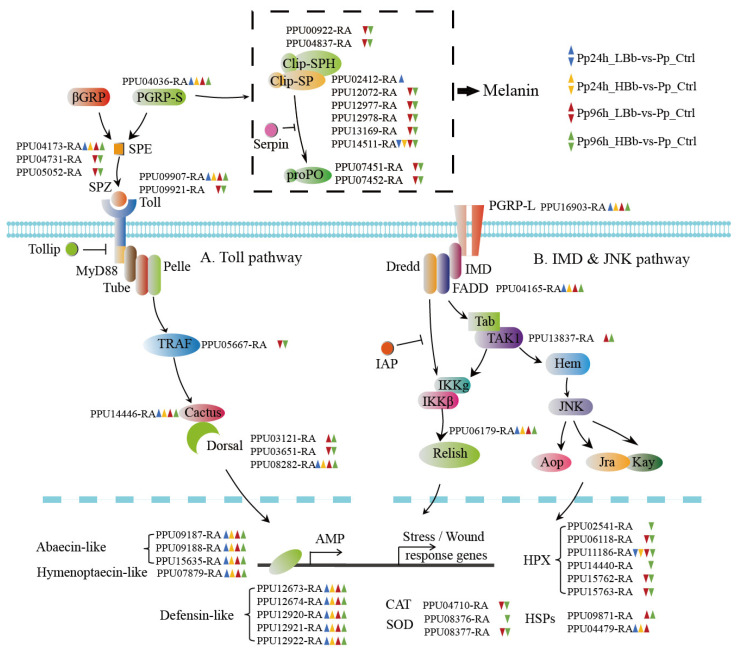
Detailed information of differentially upregulated and downregulated genes in putative immune signaling pathways and melanotic processes in *Pteromalus puparum*.

Our comprehensive analysis of immune-related genes in response to different stages and concentrations of *B. bassiana* infection in *P. puparum* provides valuable insights into the host’s immune defense mechanisms and the intricate interplay between the parasitoid and the fungal pathogen (Table S5). The following are some key findings from our analysis:

1. Differential expression of immune genes involved in recognition molecules: Several immune-related genes involved in recognition molecules showed differential expression in response to fungal infection, including scavenger receptors (*PpSCRBs*), peptidoglycan recognition proteins (*PpPGRPs*), and C-type lectins (*PpCTLs*). Most of these genes exhibited reduced expression levels after pathogen challenge.
2. Toll and IMD pathway activation: The expression levels of genes involved in the Toll and IMD immune signaling pathways, such as *PpTollB*, *PpSPZ4*, *PpCactus*, *PpDorsal1*, and *PpDorsal3*, associated with the Toll pathway, and *PpRelish*, *PpFADD*, and *PpTAK*, which participated in the IMD pathway, were enhanced during both early and late stages of infection (Figure 4). This indicates the activation of these pathways in response to *B. bassiana*.
3. Upregulation of AMP genes: Genes encoding hymenoptaecin-like, defensin-like, and abaecin-like AMP*s* were significantly upregulated in response to infection (Figure 4). Hymenoptaecin-like genes exhibited a sharp induction upon infection, with a fold change ranging from 4.5 to 8.1.
4. Involvement of autophagy: The upregulation of *PpFIP200-1*, a gene involved in autophagy, suggests the importance of autophagy in the host’s defense against fungal pathogens.
5. Serine proteases (SPs) and serine protease homologs (SPHs): In total, 55 SPs and 15 SPHs were identified among the differentially expressed genes. These genes showed enrichment in both upregulated and downregulated GO terms. We also observed a significant reduction in DEGs related to clip domain SPs/SPHs, particularly in the late infected stage, indicating the impairment of the prophenoloxidase pathway (Figure 4).
6. Downregulation of antioxidant enzymes: Genes associated with antioxidant enzymes, including SODs, catalase and HPXs, were downregulated in the late stage of infection, possibly indicating a reduced antioxidant defense response.

Overall, our study highlights the complexity of the host-parasite interaction, emphasizing the multifaceted immune responses employed by *P. puparum* to combat *B. bassiana* infection.

### 2.6. Differentially Expressed Cuticular Genes

Our analysis of cuticular and chitinase genes in response to *B. bassiana* infection in *P. puparum* reveals important insights into how the parasitoid’s cuticle and chitin-related processes are affected by the fungal pathogen (Table S5). First, among the 82 analyzed cuticular genes, 22 showed differential expression in at least one infected sample. These differentially expressed cuticular genes belong to six subfamilies: Apidermin, CPAP3, CPR-RR-UC, TWEEDLE, CPR-RR1, and CPR-RR2. Notably, the expression levels of these cuticle genes were reduced upon infection, suggesting that fungal infection disrupts cuticle integrity. Secondly, the analysis revealed the presence of seven differentially expressed genes encoding chitinase-like proteins in response to *B. bassiana* infection. Three of these genes were downregulated, while four were upregulated. Interestingly, the upregulated chitinase-like genes had significantly higher FPKM values compared to those of the downregulated ones, resulting in an overall increase in the expression profiles of chitinase genes. The elevated expression of chitinase genes suggests an accelerated degradation of chitin in the cuticle. Chitin is a major component of insect exoskeletons and fungal cell walls, so its degradation likely enables the successfully invasion of fungal spores into host insects.

Together, our findings provide evidence that *B. bassiana* infection leads to significant alterations in the expression of cuticular proteins and chitinase-like genes in *P. puparum*. Understanding these changes in cuticle integrity and chitin degradation processes sheds light on the host’s ability to combat fungal infections and adapt to environmental challenges.

### 2.7. Differentially Expressed Detoxification Genes

The analysis of detoxification-related genes in response to *B. bassiana* infection in *P. puparum* reveals several important findings regarding the host’s detoxification mechanisms and how the fungus may counteract them (Table S5).

1. Glutathione S-transferases: Out of the 20 GSTs identified in *P. puparum*, 7 were found to be differentially expressed in at least one infected sample. Remarkably, all of these differentially expressed GST genes exhibited downregulated expression upon infection. Glutathione S-transferases are known to play a crucial role in the detoxification of xenobiotics, and their reduced expression may signify a disruption in the host’s detoxification processes.
2. Cytochrome P450s: In total, 61 differentially expressed genes were annotated to the P450 family in the infected samples. The majority of these P450 genes exhibited significantly reduced expression levels following infection. Cytochrome P450 enzymes are essential for the metabolism of various toxic compounds, and their downregulation suggests a potential impairment of detoxification pathways.
3. ATP-binding cassette transporters: ATP-binding cassette transporters are known to be involved in the transport of xenobiotics and other molecules across biological membranes. Briefly, 15 out of 20 identified ABC transporters showed decreased expression after infection. This downregulation may affect the host’s ability to transport and eliminate toxic substances.
4. Carboxylesterases: Carboxylesterases are enzymes involved in the hydrolysis of ester bonds and are associated with detoxification processes. Five out of six identified CarEs exhibited reduced expression in response to infection. Similarly to the case for GSTs, P450s, and ABC transporters, the downregulation of CarEs suggests a potential compromise in detoxification pathways.
5. Late-stage downregulation: It is noteworthy that most of these detoxification-related genes were differentially downregulated during the late stages of infection. This temporal pattern may indicate that the fungus has evolved mechanisms to suppress the host’s detoxification responses as the infection progresses.

In short, we found that *B. bassiana* infection in *P. puparum* leads to the downregulation of key detoxification-related genes. This could be a strategy employed by the fungus to evade the host’s detoxification processes and establish a persistent infection. Understanding these interactions between the parasitoid and the fungal pathogen sheds light on the complex molecular mechanisms underlying insect-fungus interactions.

### 2.8. Differentially Expressed Genes in Olfactory Perception

**Figure 5:**
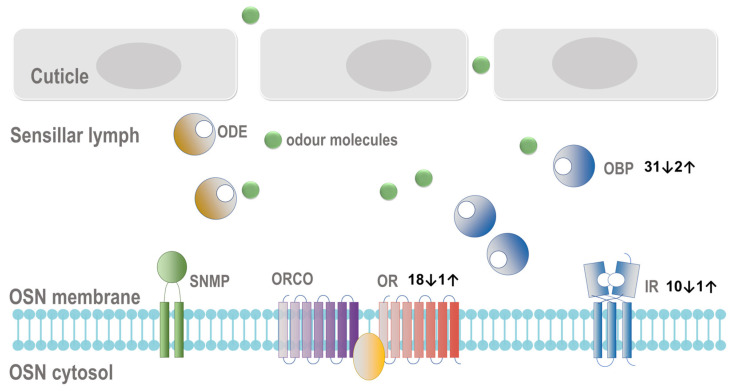
Genes involved in olfactory signaling. The number of differentially upregulated and downregulated genes related to receptors or proteins in *Pteromalus puparum* are illustrated. OBP: odorant-binding protein; OR: odorant receptor; ORCO: co-receptor of OR; IR: ionotropic receptor; SNMP: sensory neuron membrane protein; ODE: odorant-degrading enzyme.

Changes in genes related to the olfactory mechanism in response to *B. bassiana* infection in *P. puparum* have also been identified (Table S5; Figure 5).

1. Odorant-binding proteins: In total, 33 DEGs related to OBPs were identified in response to fungal infection. Interestingly, the majority of these OBP genes (31 out of 33) exhibited downregulated expression upon infection. Odorant-binding proteins play a crucial role in transporting chemical odorants to olfactory receptors, and their downregulation suggests a potential disruption in the olfactory signaling pathway.
2. Odorant receptors (ORs): We also identified 11 DEGs annotated as OR genes in response to fungal infection. Similar to OBPs, the majority of these OR genes (10 out of 11) showed downregulated expression upon infection. Odorant receptors are integral components of the olfactory receptor complex and are responsible for detecting specific odorants.
3. Ionotropic receptors (IRs): Additionally, 19 DEGs were annotated as IR genes in response to infection. Of these, 18 IR genes exhibited downregulated expression upon fungal infection. Ionotropic receptors are another class of olfactory receptors involved in detecting various chemical compounds.

These findings indicate that the parasitoid’s olfactory system undergoes significant changes in gene expression upon fungal infection, primarily characterized by the downregulation of key olfaction-related genes. This alteration in olfactory gene expression may impact the parasitoid’s ability to detect environmental cues and respond to odorants, which could have implications for its foraging and host-seeking behavior. Understanding these changes in olfactory genes adds another layer of insight into the complex interactions between the parasitoid and the fungal pathogen.

### 2.9. Validation by rt-qPCR

**Figure 6:**
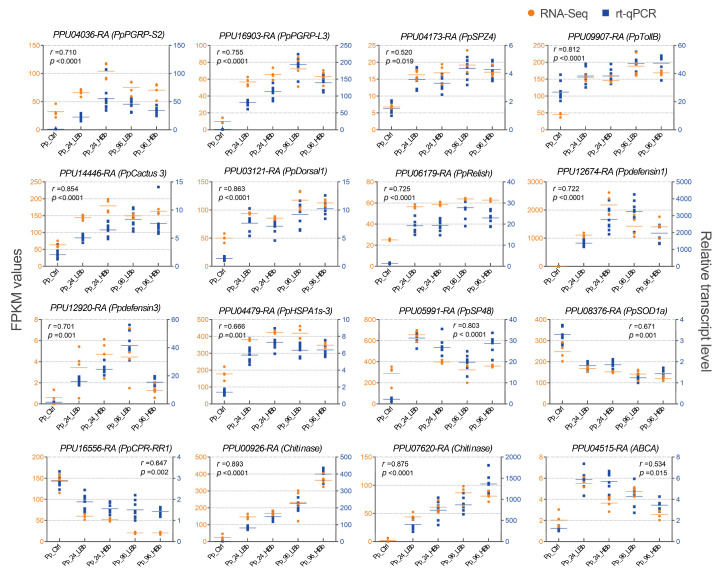
Comparison of the relative expression profiles of genes via rt-qPCR and RNA-Seq. The left vertical axis coordinate is the FPKM of RNA-Seq (orange); the right vertical axis coordinate is the relative expression level determined via rt-qPCR (blue). *r*-values are the correlation coefficients between rt-qPCR and RNA-Seq. *p*-values show the significance of the correlation coefficients.

We conducted both RNA-Seq and rt-qPCR analyses to validate the gene expression changes observed in response to *B. bassiana* infection in *P. puparum*. In total, sixteen DEGs from the genes mentioned in Table S5 were selected for the computation of their Cq values, with a focus on genes associated with immune pathways (Figure 6). Furthermore, we conducted a randomized selection of eight genes from the total *P. puparum* genes for the purpose of rt-qPCR validation (Figure S2). The correlation coefficients between the rt-qPCR and the RNA-Seq results were calculated. Among these 24 genes, we observed that 9 genes displayed extremely strong correlation coefficients (>0.8), indicating excellent agreement between the RNA-Seq data and rt-qPCR results for these genes. Additionally, twelve other genes exhibited strong correlation coefficients ranging from 0.6 to 0.79 (*p* < 0.05). To visualize the agreement between the RNA-Seq and rt-qPCR data, a scatter plot depicting the expression patterns of the selected genes was created. Overall, our study’s validation efforts indicate that the RNA-Seq results are consistent and reliable in reflecting the changes in gene expression in response to *B. bassiana* infection in *P. puparum*. This strengthens the confidence in the accuracy of the gene expression profiles obtained through RNA-Seq analysis. (Figure 6 and Figure S2).

## 3. Discussion

The limited research on the pathogenicity of *B. bassiana* to *P. puparum* highlights the need for a better understanding of the interactions between these two important species in IPM programs. Previous studies have shown that insects can be infected with fungi upon contact with fungal conidial suspensions either through the epidermis or gut, and the mortality of insects is both dose- and time-dependent [14,34,35,36]. These studies coincide with our findings that *B. bassiana* caused the death of *P. puparum* in a dose-dependent manner through both spraying and oral ingestion. Importantly, it should be noted that the susceptibility of parasitoids may differ between laboratory conditions and field settings. Previous research has suggested that the susceptibility of the aphid endoparasitoid *Aphidius colemani* to *B. bassiana* is high in laboratory settings, whereas lower infection rates are observed in greenhouse environments, likely due to complex environmental conditions [37]. Additionally, more studies have reported an additive effect rather than a reduced effect when these two biocontrol agents are combined [38,39]. Our results demonstrate that a spray of four conidia/mm^2^ or the ingestion of 3 × 10^4^ conidial/mL has an adverse effect on the survival rates of adult wasps. However, the translation of these findings into practical applications necessitates a comprehensive consideration of the complexities inherent in field environments. These complexities can be attributed to multifaceted ecological factors, notably including temperature and humidity fluctuations, food availability, and the presence of natural enemies. Consequently, the determination of appropriate application concentrations and timing in practical use remains contingent upon robust field data. Therefore, further investigations should be conducted in the interactions between *P. puparum* and *B. bassiana* in semi-field and real-world field conditions to ascertain the recommended concentration for optimized IPM practices, and ensure the maximal efficacy of *B. bassiana*-based IPM strategies.

In this study, we conducted a comprehensive analysis of the transcriptional responses of *P. puparum* upon infection with *B. bassiana* to obtain deeper insights into their interactions. Principal coordinate analysis indicated that the duration of infection had a more significant impact on gene expression levels compared to the fungal dose used. Additionally, Venn diagram analysis revealed coherent expression patterns of DEGs in all infected samples. Notably, the number of downregulated DEGs was higher than the number of the upregulated ones in all infected samples, indicating a suppression of gene expression in response to fungal infection. Previous studies have indicated that the proportion of downregulated DEGs can be influenced by various factors, including the duration of infection and the virulence of fungal strain used. For example, the downregulated DEGs were higher in number than the upregulated DEGs were in locusts 24 to 72 h post-infection (p.i.) and in *Plutella xylostella* exposed to low-virulence *B. bassiana*. However, the ratio of upregulated to downregulated DEGs was reversed when the duration of infection was shorter (i.e., 3–12 h p.i.) or when exposed to a strain of *B. bassiana* with low virulence [40,41,42]. The potent pathogenicity of the wild-type *B. bassiana* strain used in this study likely contributed to the higher number of downregulated DEGs observed in *P. puparum*. Moreover, the downregulation of genes in the expression profiles as the infection time increased further supports the idea that *B. bassiana* infection profoundly affects the transcriptional responses of *P. puparum*. Furthermore, the validation of the RNA-Seq data using rt-qPCR confirmed the reliability and accuracy of the transcriptome analysis, thereby enhancing confidence in the gene expression profiles obtained from the RNA-Seq experiment.

The innate immune system of insects plays a crucial role in recognizing and eliminating entomopathogenic fungi, thus acting as the most important defense strategy against these pathogens [12]. In this study, the Toll and IMD pathways, which are immune-related signaling pathways, were found to have a dominant impact on defending against fungal infection in *P. puparum*. Among the ten genes involved in the Toll pathway, and the three genes in the IMD pathway that showed differential expression, half of the genes in the Toll pathway and all the genes in the IMD pathway were upregulated upon infection with *B. bassiana*. Similar findings have been observed in other insects, such as honeybees and Japanese pine sawyer beetles, where immune-related pathways were upregulated in response to fungal infection [43,44]. The knockdown of key genes in the Toll and IMD pathways, including Toll, Traf, Myd88, or NF-κB transcription factors, has been shown to impair the lifespan of the hosts, further confirming the importance of these immune signaling pathways in defense against fungal invasion [15,45]. Additionally, AMPs are small proteins that are evolutionarily conserved and play a critical role in the insect defense response to fungal infection [46,47,48]. The effects of AMPs on resistance to fungi have also been confirmed by directly silencing their expression profiles [49,50,51]. Defensin and abaecin have been shown to be regulated via both Toll and IMD signaling pathways, whereas hymenoptaecin is regulated via the IMD pathway [52,53]. Upon fungal infection in *P. puparum*, we observed highly induced expression profiles of AMPs, which were consistent with the elevated expression of genes related to immune pathways. This indicates a rapid and sharp immune response to fungal infection. The regulation of AMPs by both Toll and IMD signaling pathways further emphasizes their importance in immune defense against fungi. Moreover, upregulated HSPs were found in *P. puparum* adults upon infection with *B. bassiana*, highlighting their involvement in the immune response to fungal infection. Heat shock proteins can participate in signal transduction through the Toll pathway, further indicating their role in the immune defense mechanism [16,54].

Serine proteases and their homologs are involved in both nutrient digestion and humoral immunity [55,56]. In the experiment, SPs/SPHs were significantly enriched in both upregulated and downregulated GO terms. While some of the SPs and SPHs may not be directly involved in combating fungi, it is suggested that wasps utilize digestion to provide essential elements for immunity against fungal infections [57]. The proteolytic activation of prophenoloxidase is mediated by SP cascades containing a clip domain [55,58]. The downregulation of certain SP, SPH, and PPO genes during the late infection stages may be attributed to the exhaustion of components in the melanization pathway.

Regarding antioxidant mechanisms, the enzymatic activity of SODs, HPXs, and CATs initially increased, followed by a decreasing trend in *B. bassiana*-infected *Spodoptera frugiperda* and *Spodoptera litura* [59,60]. Previous studies have suggested that various entomopathogenic fungi can induce oxidative stress through insect infection, leading to a reduction in the enzymatic activities of SODs, HPXs, and CATs. This reduction in enzymatic activities can compromise its antioxidant defense capacity and increase the probability of infection with pathogenic fungi.

The insect cuticle is a complex structure with various layers, and cuticular protein genes play a critical role in providing antifungal properties to the adult cuticle [18]. The knockdown of cuticular protein genes in beetles displayed a drastic disruption of the host defense against *B. bassiana* and *M. anisopliae* [61]. On the other hand, the upregulation of cuticle genes led to the accumulation of melanin [62]. These processes contributed to the hardening and darkening of the cuticle, which are essential defense mechanisms against fungal invasion [63,64]. In the case of *P. puparum*, the expression profiles of cuticle genes were reduced, while the expression levels of chitinase-like genes were increased. This led to an accelerated degradation of the cuticle, which may have contributed to the disruption of the cuticle upon fungal infection. The presence of melanin in *P. puparum* was suggested to positively correlate with the expression of cuticle protein genes. However, in response to fungal infection, the observed decline in the expression of genes involved in melanization and cuticle proteins indicates that the fungal spores successfully breached the cuticle and reached the hemolymph within 24 h p.i. This breach of the cuticular defense system triggers an immune system-dominated response.

The observed decline in the expression of OBPs, IRs, and ORs in *P. puparum* indicates that infection with the entomopathogenic fungus led to a reduction in the sensitivity of the olfactory system. This downregulation of olfactory genes suggests that the insect’s ability to detect and respond to odors is compromised during fungal infection. The cessation of or a reduction in olfactory signaling via the removal of odorants is an important aspect of sensory perception in insects. Odorant-degrading enzymes (ODEs), such as P450s, are implicated in the breakdown and degradation of odorants, contributing to a reduction in olfactory signaling [65]. In previous studies, it has been shown that in certain insects, the upregulation of OBPs upon fungal infection negatively affected the immune responses of the insects. This upregulation of OBPs resulted in a reduction in the expression of Toll-pathway related genes and AMPs, ultimately benefiting the successful invasion of the fungal pathogen [26]. However, in the case of *P. puparum*, the observed responses were different. Instead of upregulating OBPs, the immune genes were upregulated, and the genes involved in the olfactory system were downregulated upon fungal infection. This contrasting response suggests that there is a complex crosstalk between olfaction and immunity in *P. puparum* during fungal infection. The exact mechanisms of this crosstalk between olfaction and immunity in response to fungal infection in *P. puparum* are not yet fully understood and warrant further investigation. Understanding how the olfactory system and immune system interact and influence each other during fungal infection could provide valuable insights into the insect’s defense strategies and potential targets for biocontrol strategies.

The downregulation of detoxification genes, including GSTs, P450s, ABC transporters, and CarEs, in *P. puparum* upon fungal infection indicates a potential impairment of the insect’s classical detoxification mechanisms. These detoxification-related genes are crucial in the metabolism and elimination of various natural and environmental pollutants, as well as toxic secondary metabolites produced by pathogens [10,59,66]. The regulation of detoxification genes can vary depending on the specific fungal strains and the duration of fungal infection. Some fungi have been shown to actively enhance their virulence by reducing the expression of detoxification genes in their insect hosts. This manipulation could be a strategy employed by the pathogen to disrupt the insect’s conventional detoxification mechanisms, making it more susceptible to infection and facilitating the success of fungal invasion [67]. A similar downregulation of detoxification genes has been observed in other insect-fungi interactions, such as in *Drosophila melanogaster* [22]. This suggests that the phenomenon of the fungal manipulation of insect detoxification mechanisms might be a widespread strategy among various insect-fungus interactions [10]. The observed downregulation of detoxification genes in *P. puparum* indicates an impairment of the classical insect detoxification mechanisms, which could favor the fungal pathogen’s invasion and subsequent colonization within the insect host. However, it is important to note that the exact mechanisms and implications of this downregulation need further investigation to fully understand the dynamics of the host-pathogen interaction.

## 4. Materials and Methods

### 4.1. Fungal Strain Culture and Insects Rearing

*Pteromalus puparum* serves as the predominant parasitic species in the regulation of the small white butterfly, *Pieris rapae*, a significant agricultural pest affecting crucifer and caper families globally [68]. Therefore, *P. rapae* was selected as the host of *P. puparum* in this study. The laboratory-reared *Pieris rapae* and *P. puparum* were maintained under controlled conditions at 25 ± 1 °C and 80% relative humidity, with a photoperiod of 10 h:14 h (light: darkness). *Pieris rapae* larvae were reared on cabbage leaves until pupariation. Subsequently, the pupae were placed into sterile finger-type tubes with two-day-old mated female wasps for parasitization. After 24 h, the pupae were kept under the same environmental conditions until the emergence of wasp offspring. The emerged wasps were then transferred to new tubes with a 20% (*v*/*v*) honey solution.

Wild-type *B. bassiana* ARSEF 2860 was cultured and maintained on Sabouraud dextrose agar (SDAY) medium at a 25 ± 1 °C for 7 days in the dark, following the protocol previously described [69]. In brief, the mycelia were removed, and harvested spores were filtrated and dried for further use. To assess the spore germination rate of each batch of *B. bassiana* conidia, we measured viability by counting germinated conidia in a 24 h liquid culture containing 2% sucrose and 0.5% peptone. Conidia with a germination rate exceeding 90% within 24 h of incubation were selected for further experiments [70]. Conidia were suspended in sterile PBS with 0.05% Tween-80 for the spraying experiment and additional 20% sucrose for the oral ingestion experiment. The concentration of conidia was determined using a Neubauer counting chamber (Qiujing, Shanghai, China), and adjusted to an initial concentration of 1 × 10^8^ conidia/mL before dilution.

### 4.2. Treatment of P. puparum with B. bassiana

In this study, female adults of *P. puparum* were inoculated with *B. bassiana* conidia via two methods: the oral intake of sucrose water containing suspending conidia or surface spraying with suspending conidia. The concentration of conidia was determined using a Neubauer hemocytometer. For the feeding experiment, newly emerged female adult parasitoids were starved for 12 h and then fed with different concentrations of conidia (3 × 10^4^, 3 × 10^5^, 3 × 10^6^, 3 × 10^7^ and 3 × 10^8^ conidia/mL) for an additional 12 h. Afterwards, they were supplied with sterile sucrose water until their death, and their survival rates were recorded. A control group of parasitoids not exposed to any fungi was also included in the study.

In the spraying exposure assay, the female wasps were anesthetized with carbon dioxide for five minutes using Wall-Mount Flowbuddy Complete (Genesee Scientific, El Cajon, CA, USA) and then carefully placed in sterile 90 mm Petri dishes. Three glass cover slips (20 × 20 mm) were also included in the Petri dish alongside the wasps. Each Petri dish with parasitoids was then subjected to either a spray of the spore suspension or a control solution (0.05% Tween-80) using a handheld Micro Ulva fogger. Subsequently, the parasitoids were transferred to finger-type plastic tubes with 20% honey water (*v*:*v*). The concentration of deposited conidia was checked by counting the number of conidia/mm^2^ using microscopic counts from three glass cover slips placed alongside the wasps during spraying [71,72]. The concentration of deposited conidia used in this experiment was 500, 100, 20, 4 and 1 conidia/mm^2^. In cases where direct counting for lower concentrations of four and one conidia/mm^2^ was not accurate, they were sprayed directly after dilution based on the high concentration ratio. Adult parasitoids exposed to sterile PBS were set as control groups. After exposure, the parasitoids were transferred to finger tubes containing sterile sucrose water, and their survival rates were monitored for up to 10 days.

### 4.3. RNA Sample Preparation and Extraction

*Pteromalus puparum* adults that were exposed to *B. bassiana* via the surface spraying exposure assay were selected for further experiments. To determine the changes in the gene expressions of the parasitoids in response to the fungi, wasps were collected at two time points, 24 and 96 h after fungal treatment, using two different concentrations of conidia: 500 conidia/mm^2^ and 20 conidia/mm^2^. The collection at 24 h represented the early infection stage, while the collection at 96 h represented the late infection stage. The treated samples were named as follows: Pp_24_LBb (*P. puparum* infected with 20 conidia/mm^2^ *B. bassiana* for 24 h), Pp_24_HBb (*P. puparum* infected with 500 conidia/mm^2^ *B. bassiana* for 24 h), Pp_96_LBb (*P. puparum* infected with 20 conidia/mm^2^ *B. bassiana* for 96 h), and Pp_96_HBb (*P. puparum* infected with 500 conidia/mm^2^ *B. bassiana* for 96 h). Uninfected adult wasps were used as control groups and named as Pp_Ctrl. To account for the high mortality rates observed in insects after 96 h of infection, an excess number of parasitoids were prepared prior to infection to ensure adequate collection. Additionally, the vitality of insects was assessed visually before sampling. Those exhibiting signs of diminished vigor, such as the inability to fly or move, were excluded from the sample pool. To prepare the samples for RNA extraction, the entire body of each parasitoid was homogenized and extracted using TRIzol^®^ Reagent (Sigma-Aldrich, St. Louis, MO, USA) following the manufacturer’s protocol. We then reverse transcribed 1 μg of the total RNA into cDNA using a PrimeScript™ II first strand cDNA synthesis kit (Takara, Tokyo, Japan) for rt-qPCR analyses. Each sample included a pool of twenty individual parasitoids, and four replicates were obtained for each infected and uninfected sample.

### 4.4. Illumina Sequencing and DEG Analysis

The total amounts and integrity of the RNA samples were measured using the Bioanalyzer 2100 system (Agilent, Santa Clara, CA, USA) and Nano 6000 Assay Kit (Agilent, CA, USA). Subsequently, the cDNA libraries were generated at the Novogene sequencing center (Beijing, China). The process involved mRNA isolation using oligo(dT) magnetic beads, followed by 1st and 2nd-strand cDNA synthesis using M-MuLV Reverse Transcriptase, DNA polymerase I, dNTPs, and random hexamer primers. The libraries generated underwent selective PCR amplification and were sequenced on the NovaSeq 6000 platform (Illumina, San Diego, CA, USA). The raw sequence data were preprocessed into clean data using fastp software [73]. Paired clean reads were then aligned to the relevant reference genome using Hisat2 [74]. We used FeatureCounts to quantify the number of reads mapped to each gene, and their read count values were calculated [75]. Subsequently, FPKM values were calculated using the R package DESeq2 [76].

The annotation of *P. puparum* related to immune genes, cuticle genes, SPs and their homologs, and GSTs was readily available for DEG analysis [32,33,77,78]. The annotation of other DEGs was obtained from Pfam and Swissprot databases [79,80].

### 4.5. Validation for RNA-Seq Analysis

For the validation of RNA-Seq data, 24 DEGs were selected, and primers were designed using Primer3 [81]. *Pteromalus puparum 18S* rRNA served as an internal control, and the primer sequences can be found in Table S6. The cDNA samples, diluted at a ratio of 1:20, were used as templates for rt-qPCR analyses. These analyses were performed using iTaq™ Universal SYBR^®^ Green Supermix (Bio-Rad, Hercules, CA, USA) on Bio-Rad Real-Time PCR System (Bio-Rad, Hercules, CA, USA). The rt-qPCR conditions consisted of an initial denaturation step at 94 °C for 3 min, followed by 39 cycles of 94 °C for 30 s, and 60 °C for 30 s. To assess non-specific amplification, melting curve analysis was performed. The 2^−ΔΔCT^ method was utilized to calculate the relative expression levels of these target genes [82].

### 4.6. Data Analysis

To test the survival rates and LT50 of parasitoids subjected to fungal treatment, Kaplan-Meier survival analysis was employed [83]. Pairwise multiple comparison was performed using the Holm-Sidak method to determine significant differences between different treatments.

The read count values of all genes were obtained and used to identify DEGs via DESeq2 with a statistical significance threshold of |log_2_ (infected group/control group)| ≥ 1 and a *p*-value of < 0.05. To gain further insight into the biological functions and metabolic pathways associated with the DEGs, GO and KEGG pathway analyses were conducted. Significant enriched GO terms were selected with a *p*-value < 0.05 and KEGG pathways were identified using a *p*-value of < 0.05.

Each experiment for rt-qPCR analyses was repeated at least three times to ensure reproducibility. Statistical analyses were conducted using Student’s *t*-test, and a *p*-value of <0.05 was considered a significant difference.

## 5. Conclusions

**Figure 7:**
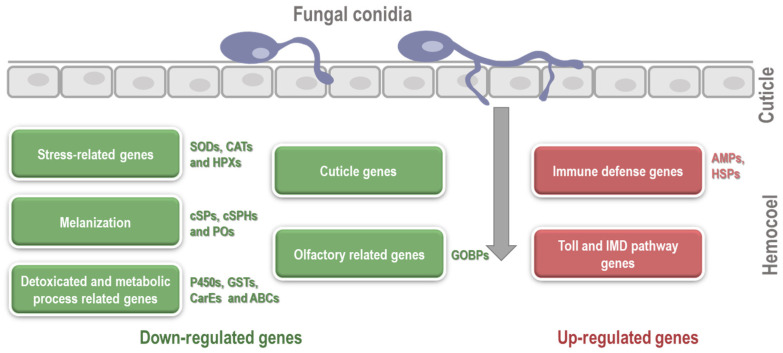
A summary of altered gene expression levels upon *beauveria bassiana* infection in the parasitoid *Pteromalus puparum*.

Our study provided valuable insights into the interactions between *P. puparum* and *B. bassiana*, two important species in IPM programs. We discovered that *B. bassiana* significantly affects the survival rates of *P. puparum*, both through direct spraying and oral ingestion. Through RNA-seq analysis, we identified the crucial role of the insect’s immune defense mechanisms in protecting against fungal invasion. Genes involved in AMPs, HSPs, and Toll and IMD immune pathways were upregulated, indicating an active immune response to fungal infection (Figure 7). Conversely, we observed differential downregulations in gene expression related to stress response, melanization, detoxication, and cuticle formation (Figure 7). This suggests that the conventional defense mechanisms of *P. puparum*, such as stress tolerance, melanin-based immunity, detoxification, and cuticle strength, are compromised in response to fungal infection. The complex defense responses of *P. puparum* to *B. bassiana* highlight the dynamic nature of the host-pathogen interaction. Understanding these interactions is crucial for developing effective pest management strategies under field conditions. By obtaining a deep understanding of the immune responses and vulnerabilities of *P. puparum* to *B. bassiana*, we can improve the efficacy of biological control measures and the sustainability of pest management practices. Overall, this study contributes to our knowledge of insect-fungus interactions and lays a foundation for further research on the molecular mechanisms underlying the immune responses and susceptibility of insects to entomopathogenic fungi. Such research is essential for the development of targeted and efficient approaches in pest control and crop protection.

## Data Availability

Data are contained within the article.

## Supplementary Materials

The Supplementary Materials are available online at https://www.mdpi.com/article/10.3390/ijms242317030/s1.
