# Supplementary material for: Altered Gene Expression of the Parasitoid Pteromalus puparum after Entomopathogenic Fungus Beauveria bassiana Infection
Source: Int J Mol Sci. 2023 Dec 1;24(23):17030. doi: 10.3390/ijms242317030 (PMC10707577; doi:10.3390/ijms242317030)
Supplement: Supplementary file 1 [file ijms-24-17030-s001.zip › ijms-2706618-supplementary.pdf]

Supplementary Figures and Tables

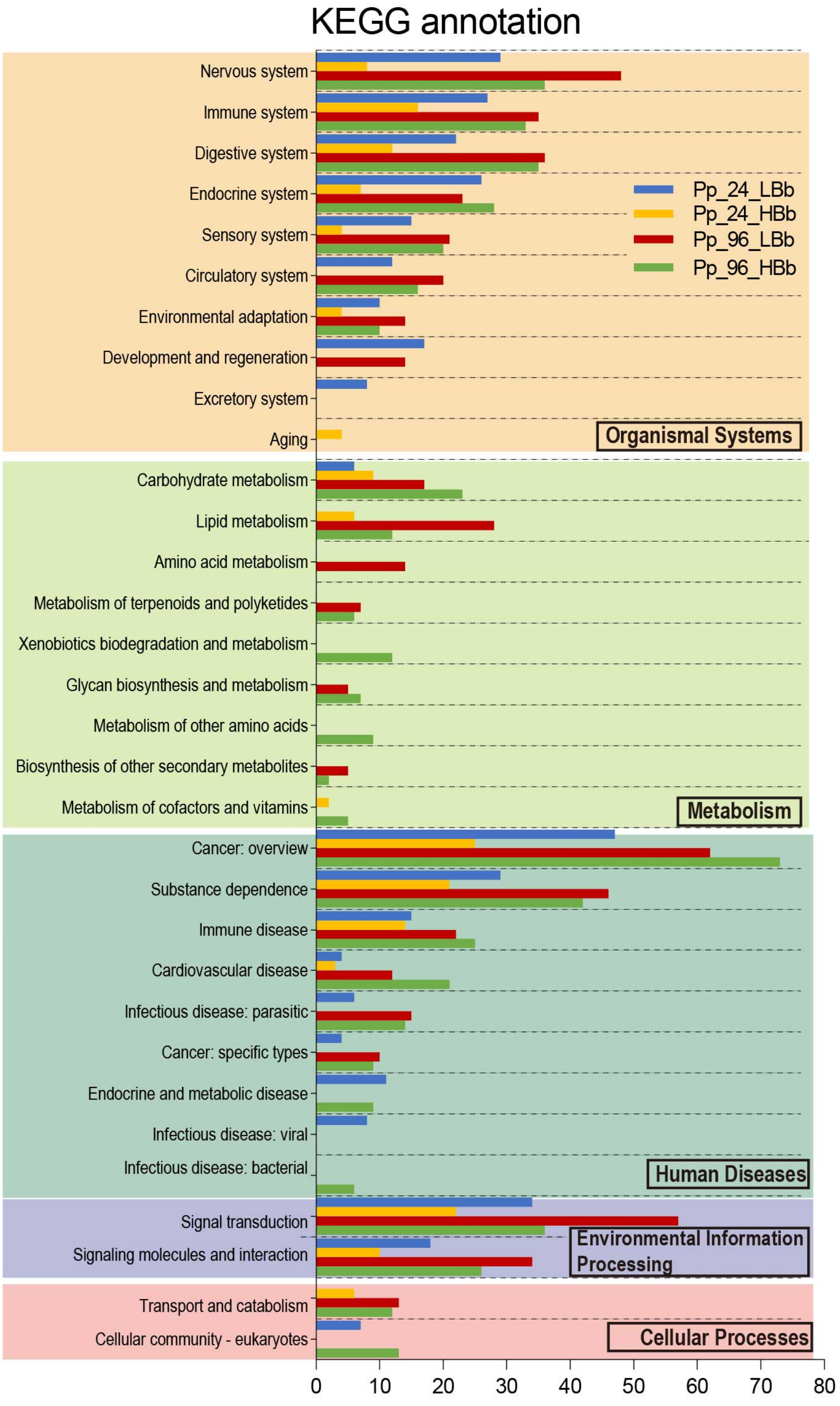

Figure S1. KEGG enrichment analysis of the DEGs from HBb- and LBb-treated *P. puparum* in both 24h and 96 h post infection. p-value < 0.05 was used as the significance threshold.

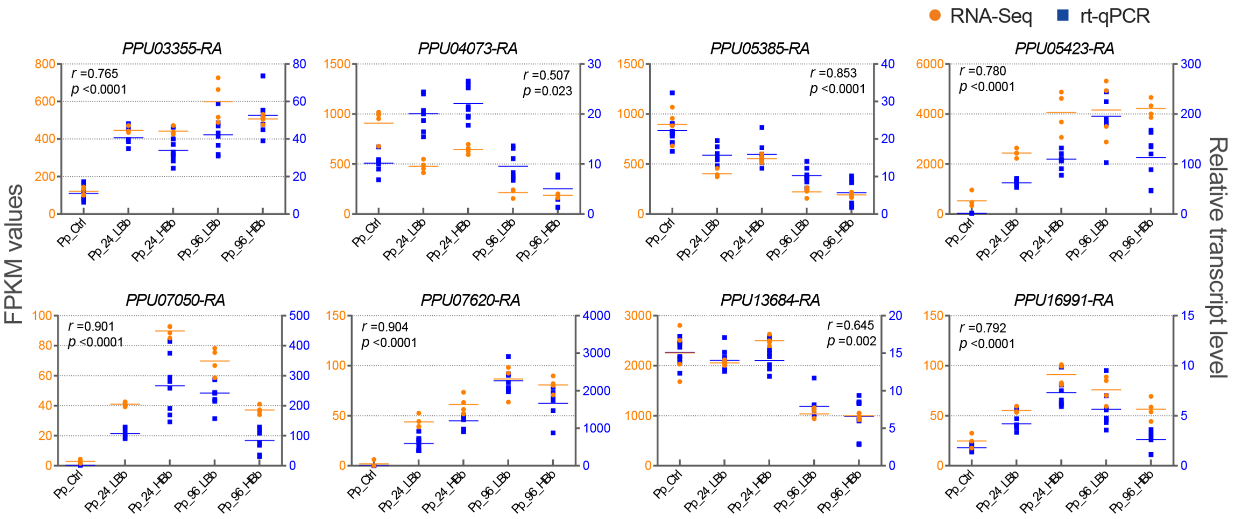

Figure S2. Comparison of the relative expression profiles of genes by rt-qPCR **and RNA-Seq.**

Left vertical axis coordinate is FPKM of RNA-Seq (orange); right vertical axis coordinate is relative expression level of rt-qPCR (blue). *r*-values are the correlation coefficients between rt-qPCR and RNA-Seq. *p*-values show the significance of the correlation coefficients.

Table S1. A summary of RNA sequencing and mapping using the *Pteromalus puparum* and *Beauveria bassiana* genome as the reference. Columns represent number of raw sequencing reads, number of raw bases, number of clean reads, number of clean bases, error rate, GC content, percentage of bases with Phred values score more than 20 or 30 to total bases, and ratio of sequences mapped to *P. puparum* and *B. bassiana* genome.

Samples	Raw_reads	Raw_bases	Clean_reads	Clean_bases	Error_rate	GC_pct	Q20	Q30	Total_mapping to *P. puparum*(%)	Total_mapping to *B. bassiana*(%)
Pp_Ctrl_1	4.70E+07	7.04G	4.40E+07	6.61G	0.03	45.23	97.89	94.08	95.98	/
Pp_Ctrl_2	4.55E+07	6.82G	4.27E+07	6.4G	0.03	44.2	97.7	93.55	95.45	/
Pp_Ctrl_3	4.63E+07	6.94G	4.35E+07	6.52G	0.03	44.57	97.76	93.72	95.67	/
Pp_Ctrl_4	4.61E+07	6.92G	4.24E+07	6.36G	0.03	44.35	97.81	93.83	95.69	/
Pp_24_LBb_1	4.64E+07	6.97G	4.46E+07	6.68G	0.03	42.51	96.36	90.62	93.54	/
Pp_24_LBb_2	5.40E+07	8.09G	5.13E+07	7.7G	0.03	42.29	97.24	92.33	93.54	/
Pp_24_LBb_3	5.83E+07	8.75G	5.56E+07	8.34G	0.03	42.59	97	91.83	93.82	/
Pp_24_LBb_4	4.55E+07	6.82G	4.31E+07	6.46G	0.03	43.16	97.5	92.91	94.39	/
Pp_96_LBb_1	4.57E+07	6.85G	4.36E+07	6.54G	0.03	43.13	97.13	92.08	91.26	0.03
Pp_96_LBb_2	5.22E+07	7.83G	5.02E+07	7.54G	0.03	41.74	97.16	92.11	90.59	0.04
Pp_96_LBb_3	4.40E+07	6.61G	4.23E+07	6.35G	0.03	42.93	97.16	92.09	90.89	0.07
Pp_96_LBb_4	4.66E+07	6.99G	4.41E+07	6.62G	0.03	43.27	97.35	92.53	91.93	0.03
Pp_24_HBb_1	4.45E+07	6.68G	4.15E+07	6.23G	0.03	43.97	97.16	92.22	92.15	/
Pp_24_HBb_2	4.27E+07	6.41G	4.01E+07	6.02G	0.03	43.49	97.19	92.25	87.62	/
Pp_24_HBb_3	4.40E+07	6.6G	4.15E+07	6.22G	0.03	43.28	97.15	92.13	92.34	/
Pp_24_HBb_4	4.64E+07	6.95G	4.39E+07	6.58G	0.03	43.47	97.34	92.54	92.54	/
Pp_96_HBb_1	4.70E+07	7.05G	4.40E+07	6.6G	0.03	42.79	97.05	91.88	91.91	0.23
Pp_96_HBb_2	4.64E+07	6.96G	4.38E+07	6.56G	0.03	42.43	97.09	91.97	91.69	0.17
Pp_96_HBb_3	4.56E+07	6.84G	4.29E+07	6.44G	0.03	41.75	96.8	91.34	91.09	0.19

Pp_96_HBb_4	4.60E+07	6.89G	4.29E+07	6.44G	0.03	41.76	97.11	91.99	91.3	0.17

Table S2. The correlation coefficients of 14946 gene expression levels between different infected *Pteromalus puparum* using log2 fold changes

	Log_2 (Pp_24_HBb/Pp_Ctrl)	Log_2 (Pp_96_LBb/Pp_Ctrl)	Log_2 (Pp_96_HBb/Pp_Ctrl)
Log_2 (Pp_24_LBb/Pp_Ctrl)	0.699 (<0.0001)	0.750 (<0.0001)	0.734 (<0.0001)
Log_2 (Pp_24_HBb/Pp_Ctrl)	/	0.751 (<0.0001)	0.735 (<0.0001)
Log_2 (Pp_96_LBb/Pp_Ctrl)	/	/	0.841 (<0.0001)

Table S3. GO enrichment of differentially upregulated genes

Class	ID	Description	Pp_24_LBb		Pp_24_HBb		Pp_96_LBb		Pp_96_HBb	
			Number	q-value	Number	q-value	Number	q-value	Number	q-value
Biological Process	GO:0000270	peptidoglycan metabolic process	/	/	3	0.017	/	/	/	/
Biological Process	GO:0031497	chromatin assembly	7	0.001	8	0.000	6	0.022	6	0.023
Biological Process	GO:0006027	glycosaminoglycan catabolic process	/	/	3	0.017	/	/	/	/
Biological Process	GO:0009253	peptidoglycan catabolic process	/	/	3	0.017	/	/	/	/
Biological Process	GO:0006022	aminoglycan metabolic process	/	/	3	0.021	/	/	/	/
Biological Process	GO:0006952	defense response	4	0.001	4	0.000	4	0.008	4	0.007
Biological Process	GO:0070271	protein complex biogenesis	/	/	8	0.002	/	/	/	/
Biological Process	GO:0006334	nucleosome assembly	7	0.001	8	0.000	6	0.022	6	0.023
Biological Process	GO:0071103	DNA conformation change	7	0.010	8	0.000	/	/	/	/
Biological Process	GO:0006325	chromatin organization	8	0.003	8	0.000	8	0.022	8	0.023
Biological Process	GO:0034622	cellular macromolecular complex assembly	/	/	8	0.001	/	/	/	/
Biological Process	GO:0051276	chromosome organization	/	/	8	0.005	/	/	/	/
Biological Process	GO:0022607	cellular component assembly	/	/	8	0.013	/	/	/	/

Biological Process	GO:0043933	macromolecular complex subunit organization	/	/	8	0.029	/	/	/	/
Biological Process	GO:0044085	cellular component biogenesis	/	/	8	0.050	/	/	/	/
Biological Process	GO:0006508	proteolysis	38	0.000	29	0.000	34	0.022	/	/
Biological Process	GO:0019538	protein metabolic process	49	0.047	/	/	/	/	/	/
Biological Process	GO:0032259	methylation	/	/	/	/	5	0.029	6	0.017
Biological Process	GO:0043170	macromolecule metabolic process	/	/	/	/	90	0.011	85	0.023
Biological Process	GO:0071704	organic substance metabolic process	/	/	/	/	101	0.049	/	/
Biological Process	GO:0006807	nitrogen compound metabolic process	/	/	/	/	95	0.022	91	0.025
Biological Process	GO:0006461	protein complex assembly	/	/	8	0.002	/	/	/	/
Biological Process	GO:0065003	macromolecular complex assembly	/	/	8	0.005	/	/	/	/
Biological Process	GO:0071822	protein complex subunit organization	/	/	8	0.006	/	/	/	/
Biological Process	GO:1901136	carbohydrate derivative catabolic process	/	/	3	0.041	/	/	/	/
Cellular Component	GO:0005576	extracellular region	/	/	5	0.037	/	/	/	/
Cellular Component	GO:0000786	nucleosome	7	0.000	8	0.000	6	0.001	6	0.001
Cellular Component	GO:0044815	DNA packaging complex	7	0.000	8	0.000	6	0.001	6	0.001
Cellular Component	GO:0032993	protein-DNA complex	7	0.000	8	0.000	6	0.001	6	0.001
Cellular Component	GO:0005694	chromosome	8	0.000	9	0.000	8	0.001	8	0.002
Cellular Component	GO:0043232	intracellular non-membrane-bounded organelle	/	/	9	0.027	/	/	/	/

Cellular Component	GO:0005634	nucleus	/	/	/	/	19	0.019	22	0.002
Cellular Component	GO:0031262	Ndc80 complex	/	/	/	/	2	0.049	2	0.045
Cellular Component	GO:0005622	intracellular	/	/	/	/	/	/	39	0.034
Cellular Component	GO:0005623	cell	/	/	/	/	/	/	39	0.046
Cellular Component	GO:0043229	intracellular organelle	/	/	/	/	/	/	33	0.046
Molecular Function	GO:0008745	N-acetylmuramoyl-L-alanine amidase activity	/	/	3	0.025	/	/	/	/
Molecular Function	GO:0046983	protein dimerization activity	16	0.006	16	0.000	24	0.000	27	0.000
Molecular Function	GO:0004252	serine-type endopeptidase activity	31	0.000	25	0.000	27	0.000	22	0.020
Molecular Function	GO:0008233	peptidase activity	37	0.000	30	0.000	33	0.017	/	/
Molecular Function	GO:0046982	protein heterodimerization activity	15	0.000	14	0.000	22	0.000	26	0.000
Molecular Function	GO:0004175	endopeptidase activity	32	0.000	26	0.000	28	0.004	/	/
Molecular Function	GO:0016787	hydrolase activity	58	0.000	45	0.000	/	/	/	/
Molecular Function	GO:0003676	nucleic acid binding	/	/	/	/	62	0.007	67	0.001
Molecular Function	GO:0005515	protein binding	/	/	/	/	103	0.008	113	0.000
Molecular Function	GO:0003677	DNA binding	/	/	/	/	35	0.019	38	0.008

Table S4. GO enrichment of differentially downregulated genes

Class	ID	Description	Pp_24_LBb		Pp_24_HBb		Pp_96_LBb		Pp_96_HBb	
			Number	q-value	Number	q-value	Number	q-value	Number	q-value
Biological Process	GO:0007186	G-protein coupled receptor signaling pathway	27	0.000	16	0.002	40	0.000	/	/
Biological Process	GO:0007165	signal transduction	50	0.000	26	0.017	/	/	/	/

Biological Process	GO:0044700	single organism signaling	53	0.000	28	0.008	68	0.028	/	/
Biological Process	GO:0055085	transmembrane transport	/	/	31	0.013	100	0.000	99	0.000
Biological Process	GO:0055114	oxidation-reduction process	/	/	38	0.008	135	0.000	139	0.000
Biological Process	GO:0044765	single-organism transport	/	/	42	0.023	137	0.000	128	0.000
Biological Process	GO:0044699	single-organism process	129	0.009	114	0.000	382	0.000	352	0.000
Biological Process	GO:0006816	calcium ion transport	3	0.029	/	/	/	/	/	/
Biological Process	GO:0006813	potassium ion transport	6	0.004	/	/	/	/	/	/
Biological Process	GO:0007156	homophilic cell adhesion via plasma membrane adhesion molecules	8	0.000	/	/	/	/	/	/
Biological Process	GO:0007155	cell adhesion	10	0.000	/	/	/	/	/	/
Biological Process	GO:0035556	intracellular signal transduction	21	0.000	/	/	/	/	/	/
Biological Process	GO:0006811	ion transport	33	0.000	/	/	54	0.000	/	/
Biological Process	GO:0051716	cellular response to stimulus	50	0.000	/	/	/	/	/	/
Biological Process	GO:0050896	response to stimulus	52	0.000	/	/	/	/	/	/
Biological Process	GO:0050794	regulation of cellular process	65	0.000	/	/	/	/	/	/
Biological Process	GO:0044763	single-organism cellular process	94	0.000	/	/	/	/	/	/
Biological Process	GO:0006810	transport	/	/	/	/	138	0.000	129	0.000
Biological Process	GO:0051179	localization	/	/	/	/	140	0.000	131	0.000
Biological Process	GO:0044710	single-organism metabolic process	/	/	/	/	178	0.000	175	0.000
Biological Process	GO:0030001	metal ion transport	10	0.008	/	/	/	/	/	/
Biological Process	GO:0065007	biological regulation	68	0.000	/	/	/	/	/	/
Biological Process	GO:0007205	protein kinase C-activating G-protein coupled receptor signaling pathway	4	0.003	/	/	/	/	/	/
Biological Process	GO:1902578	single-organism localization	/	/	43	0.020	138	0.000	129	0.000
Cellular Component	GO:0005576	extracellular region	/	/	10	0.038	28	0.000	26	0.000
Cellular Component	GO:0016021	integral component of membrane	51	0.012	46	0.000	134	0.000	120	0.000

Cellular Component	GO:0044425	membrane part	/	/	46	0.000	138	0.000	120	0.000
Cellular Component	GO:0016020	membrane	96	0.000	68	0.000	217	0.000	188	0.000
Molecular Function	GO:0015276	ligand-gated ion channel activity	14	0.001	8	0.048	23	0.000	/	/
Molecular Function	GO:0008061	chitin binding	/	/	8	0.016	18	0.003	18	0.001
Molecular Function	GO:0042302	structural constituent of cuticle	/	/	8	0.047	/	/	/	/
Molecular Function	GO:0022836	gated channel activity	17	0.000	10	0.008	27	0.000	18	0.035
Molecular Function	GO:0004930	G-protein coupled receptor activity	22	0.000	14	0.001	33	0.000	24	0.009
Molecular Function	GO:0020037	heme binding	/	/	21	0.000	62	0.000	67	0.000
Molecular Function	GO:0005549	odorant binding	/	/	24	0.000	46	0.043	43	0.034
Molecular Function	GO:0099600	transmembrane receptor activity	43	0.000	28	0.003	68	0.004	/	/
Molecular Function	GO:0048037	cofactor binding	/	/	30	0.000	97	0.000	102	0.000
Molecular Function	GO:0016491	oxidoreductase activity	/	/	30	0.022	114	0.000	114	0.000
Molecular Function	GO:0022857	transmembrane transporter activity	48	0.007	39	0.000	127	0.000	117	0.000
Molecular Function	GO:0005230	extracellular ligand-gated ion channel activity	11	0.003	/	/	17	0.002	/	/
Molecular Function	GO:0005509	calcium ion binding	21	0.000	/	/	25	0.046	25	0.016
Molecular Function	GO:0015075	ion transmembrane transporter activity	34	0.000	/	/	56	0.000	/	/
Molecular Function	GO:0008194	UDP-glycosyltransferase activity	/	/	/	/	12	0.033	15	0.000
Molecular Function	GO:0016298	lipase activity	/	/	/	/	18	0.001	19	0.000
Molecular Function	GO:0050662	coenzyme binding	/	/	/	/	35	0.013	35	0.003
Molecular Function	GO:0046914	transition metal ion binding	/	/	/	/	81	0.000	79	0.000
Molecular Function	GO:0004601	peroxidase activity	/	/	/	/	/	/	8	0.003
Molecular Function	GO:0004252	serine-type endopeptidase activity	/	/	/	/	/	/	46	0.013
Molecular Function	GO:0022839	ion gated channel activity	16	0.000	9	0.025	26	0.000	/	/

Molecular Function	GO:0016705	oxidoreductase activity, acting on paired donors, with incorporation or reduction of molecular oxygen	/	/	22	0.000	58	0.000	60	0.000
Molecular Function	GO:0005506	iron ion binding	/	/	20	0.000	54	0.000	59	0.000
Molecular Function	GO:0005216	ion channel activity	32	0.000	14	0.004	43	0.000	30	0.002
Molecular Function	GO:0022891	substrate-specific transmembrane transporter activity	34	0.000	/	/	58	0.000	/	/
Molecular Function	GO:0022892	substrate-specific transporter activity	35	0.000	/	/	63	0.000	/	/
Molecular Function	GO:0015103	inorganic anion transmembrane transporter activity	34	0.000	/	/	51	0.000	/	/
Molecular Function	GO:0008509	anion transmembrane transporter activity	34	0.000	/	/	52	0.000	/	/
Molecular Function	GO:0043169	cation binding	/	/	/	/	117	0.000	117	0.000
Molecular Function	GO:0004806	triglyceride lipase activity	/	/	/	/	16	0.000	17	0.000
Molecular Function	GO:0046872	metal ion binding	/	/	/	/	113	0.000	113	0.000
Molecular Function	GO:0050660	flavin adenine dinucleotide binding	/	/	/	/	20	0.043	19	0.034
Molecular Function	GO:0005261	cation channel activity	5	0.030	/	/	7	0.047	/	/
Molecular Function	GO:0004143	diacylglycerol kinase activity	4	0.004	/	/	4	0.050	/	/
Molecular Function	GO:0022843	voltage-gated cation channel activity	2	0.038	/	/	/	/	/	/
Molecular Function	GO:0005262	calcium channel activity	3	0.012	/	/	/	/	/	/
Molecular Function	GO:0004435	phosphatidylinositol phospholipase C activity	3	0.025	/	/	/	/	/	/
Molecular Function	GO:0015085	calcium ion transmembrane transporter activity	4	0.001	/	/	/	/	/	/
Molecular Function	GO:0072509	divalent inorganic cation transmembrane transporter activity	4	0.004	/	/	/	/	/	/
Molecular Function	GO:0008081	phosphoric diester hydrolase activity	6	0.016	/	/	/	/	/	/

Molecular Function	GO:0004888	transmembrane signaling receptor activity	33	0.011	/	/	/	/	/	/
Molecular Function	GO:0004871	signal transducer activity	35	0.012	/	/	/	/	/	/
Molecular Function	GO:0015293	symporter activity	/	/	/	/	/	/	4	0.017
Molecular Function	GO:0016831	carboxy-lyase activity	/	/	/	/	/	/	7	0.041
Molecular Function	GO:0016684	oxidoreductase activity, acting on peroxide as acceptor	/	/	/	/	/	/	8	0.016
Molecular Function	GO:0019842	vitamin binding	/	/	/	/	/	/	12	0.044
Molecular Function	GO:0016614	oxidoreductase activity, acting on CH-OH group of donors	/	/	/	/	/	/	18	0.040
Molecular Function	GO:0008236	serine-type peptidase activity	/	/	/	/	/	/	47	0.025
Molecular Function	GO:0004175	endopeptidase activity	/	/	/	/	/	/	57	0.010
Molecular Function	GO:0003824	catalytic activity	/	/	/	/	/	/	351	0.012

Table S5. A list of part differentially expressed genes with important functions

Gene ID	Description	Log_2(Pp_LBb24/Pp_Ctrl)	Log_2(Pp_HBb24/Pp_Ctrl)	Log_2(Pp_LBb96/Pp_Ctrl)	Log_2(Pp_HBb96/Pp_Ctrl)
**Genes involved in immunity**					
Recognition molecules					
Scavenger receptor					
PPU03617-RA	PpSCRB1	/	/	-1.0058	-1.1335
PPU02550-RA	PpSCRB5	/	/	-1.447	-1.5815
C-type lectin					
PPU06505-RA	PpCTL2	/	/	-1.6785	/

PPU04830-RA	PpCTL3	-1.1131	-1.1772	-2.0318	-1.8379
PPU06909-RA	PpCTL4	/	-2.3043	/	/
PPU09980-RA	PpCTL7	-1.4941	-2.0532	-2.2245	-2.6885
PPU09193-RA	PpCTL16	/	/	-2.1548	-2.7628
PPU16931-RA	PpCTL17	/	/	1.1279	1.2187
PPU16930-RA	PpCTL20	-1.2511	-1.383	-2.5271	-2.8942
PPU08587-RA	PpCTL21	/	/	/	-1.4435
PPU08588-RA	PpCTL22	/	-1.2199	-2.4486	-3.4143
PPU10571-RA	PpCTL26	/	/	-1.0735	-1.0212
Peptidoglycan recognition protein					
PPU04036-RA	PpPGRP-S2	1.0397	1.6386	1.1418	1.1143
PPU16903-RA	PpPGRP-L3	2.4933	2.6445	2.763	2.6292
Immune pathway signaling molecules					
Toll pathway molecules					
PPU05052-RA	PpSPZ1	/	/	-1.0238	-1.1094
PPU04173-RA	PpSPZ4	1.2677	1.2548	1.4207	1.3156
PPU04731-RA	PpSPZ5	/	/	-1.3957	-1.4443
PPU09907-RA	PpTollB	1.7851	1.6351	1.9893	1.89
PPU09921-RA	PpTollC	/	/	-1.1283	-1.1098
PPU05667-RA	PpTraf	/	/	-1.5463	-1.3036
PPU14446-RA	PpCactus3	1.1703	1.1645	1.4057	1.3343
PPU03121-RA	PpDorsal1	/	/	1.1488	1.1494
PPU03651-RA	PpDorsal2	/	/	-2.0805	-1.3065
PPU08282-RA	PpDorsal3	1.1547	1.0058	1.4803	1.5487
IMD pathway molecules					
PPU04165-RA	PpFADD	1.6049	1.4338	1.5925	1.5318

PPU13837-RA	PpTAK1	/	/	1.0584	1.0213
PPU06179-RA	PpRelish	1.1727	1.1678	1.2703	1.3067
Immune effectors					
Antimicrobial peptide					
PPU07879-RA	Pphymenoptaecin	4.587	5.1204	5.1552	4.9964
PPU12673-RA	Ppdefensin1	6.1498	7.0987	6.684	6.7933
PPU12674-RA	Ppdefensin1	7.1892	8.102	7.47	7.5046
PPU12920-RA	Ppdefensin3	2.5245	2.9201	2.82	/
PPU12921-RA	Ppdefensin3	7.7718	8.0473	8.0717	7.9647
PPU12922-RA	Ppdefensin3	2.8302	2.4012	1.8653	1.3728
PPU09187-RA	Ppabaecin-1	/	2.7185	3.1399	2.8638
PPU09188-RA	Ppabaecin-1	/	2.4328	2.7305	/
PPU15635-RA	Ppabaecin-1	1.9368	1.7342	3.3026	1.9487
Heat shock protein					
PPU09871-RA	PpHSPA1s-3	/	/	1.7792	1.5545
PPU04479-RA	PpHSPA1s-4	1.0987	1.1896	1.1578	/
Autophagy					
PPU06071-RA	PpFIP200-1	1.5759	1.6252	1.6815	1.7274
Serine protease cascade					
PPU02412-RA	PpcSP2	1.337	/	/	/
PPU12072-RA	PpcSP7	/	/	-1.4139	-1.9186
PPU12977-RA	PpcSP11	/	/	-2.1942	-1.984
PPU12978-RA	PpcSP12	/	/	-1.0885	-1.2206
PPU13169-RA	PpcSP13	/	/	-1.0408	-1.1419
PPU14511-RA	PpcSP16	-1.1261	-1.355	-2.4874	-2.4874
PPU00922-RA	PpcSPH1	/	/	-1.1407	-1.2215

PPU04837-RA	PpcSPH2	/	/	/	-1.0732
PPU07451-RA	PpPO1	/	/	-1.755	-1.9893
PPU07452-RA	PpPO3	/	/	-1.926	-1.8385
Serine protease					
PPU00428-RA	PpSP3	7.9069	8.3443	8.1293	/
PPU00429-RA	PpSP4	3.0539	3.0985	3.3979	3.0452
PPU00430-RA	PpSP5	/	/	/	3.2039
PPU03109-RA	PpSP12	/	/	-3.2256	-4
PPU03538-RA	PpSP13	-1.861	-2.2147	-2.7873	-2.486
PPU03539-RA	PpSP14	-1.0579	-1.5477	-1.791	-1.4049
PPU03540-RA	PpSP15	2.0004	1.8397	2.1208	2.644
PPU03541-RA	PpSP16	2.7873	2.5566	2.3141	3.1747
PPU03542-RA	PpSP17	3.4465	2.97	3.58	3.681
PPU03821-RA	PpSP19	1.1485	/	1.4267	/
PPU04266-RA	PpSP22	3.2654	2.5687	1.6826	2.0393
PPU04980-RA	PpSP24	-2.296	-1.8458	-2.444	-1.638
PPU05858-RA	PpSP31	/	/	2.1255	/
PPU05933-RA	PpSP32	1.562	1.0943	1.4144	1.6692
PPU05945-RA	PpSP35	/	/	-1.2456	-1.6169
PPU05950-RA	PpSP38	/	/	-2.113	-2.0785
PPU05951-RA	PpSP39	/	/	-2.3676	-3.0039
PPU05990-RA	PpSP47	1.8136	2.1538	2.3951	2.6232
PPU05991-RA	PpSP48	1.1907	/	/	/
PPU05992-RA	PpSP49	2.8436	2.0655	1.8384	1.9142
PPU05993-RA	PpSP50	1.4194	/	/	/
PPU05994-RA	PpSP51	3.1779	2.3164	/	/

PPU05997-RA	PpSP53	2.3056	1.9043	2.5266	2.8731
PPU05998-RA	PpSP54	2.7331	/	2.7525	2.6568
PPU06001-RA	PpSP55	-1.1393	-1.3758	-1.4195	-2.12
PPU06034-RA	PpSP56	/	/	-1.3268	/
PPU06222-RA	PpSP58	/	/	-3.8709	-3.7868
PPU07067-RA	PpSP62	/	/	-2.6193	-2.5048
PPU07216-RA	PpSP63	2.6841	2.2181	/	-1.6184
PPU07259-RA	PpSP64	1.4039	1.3388	1.3701	1.6236
PPU07692-RA	PpSP67	/	/	-1.0764	/
PPU08115-RA	PpSP68	/	/	/	-2.1547
PPU08246-RA	PpSP71	/	/	/	-2.9289
PPU08497-RA	PpSP72	/	-1.1276	-2.5248	-3.2803
PPU08543-RA	PpSP73	-2.3842	-2.3384	-3.0778	-2.5396
PPU08997-RA	PpSP78	1.8331	1.7821	1.9164	2.1496
PPU09959-RA	PpSP84	-1.1638	/	-1.8637	-1.1484
PPU10521-RA	PpSP86	5.2689	4.7784	5.3122	6.1463
PPU10522-RA	PpSP87	3.734	3.2626	3.4757	3.4018
PPU10524-RA	PpSP88	3.3491	3.0625	2.1813	2.8823
PPU10525-RA	PpSP89	2.314	2.2125	1.3826	/
PPU10739-RA	PpSP90	/	-1.1087	-3.7447	-4.5967
PPU12246-RA	PpSP91	2.8755	3.9535	/	/
PPU12255-RA	PpSP99	/	/	-2.7382	-2.3617
PPU12258-RA	PpSP101	-1.3355	-1.6045	-1.8768	-2.2637
PPU12467-RA	PpSP108	/	/	1.039	1.1189
PPU12718-RA	PpSP110	/	/	/	-1.7065
PPU12955-RA	PpSP112	-1.539	-1.6281	-2.5727	-2.2855

PPU12981-RA	PpSP114	/	-1.435	-1.7489	-1.4594
PPU13016-RA	PpSP115	1.0835	1.1729	/	/
PPU13846-RA	PpSP120	1.9716	1.4549	1.3943	1.8438
PPU13886-RA	PpSP121	/	/	-1.3749	-2.1118
PPU14838-RA	PpSP123	/	-1.6774	-2.5461	-1.8281
PPU15468-RA	PpSP124	/	/	-1.6218	/
PPU15850-RA	PpSP125	4.3095	4.0941	3.9968	3.7201
Serine protease homolog					
PPU00799-RA	PpSPH1	/	/	-2.5191	-1.8606
PPU00800-RA	PpSPH2	/	/	-1.3551	/
PPU03217-RA	PpSPH5	-1.6452	-1.158	-1.5457	-1.5578
PPU05294-RA	PpSPH7	/	/	-1.6165	-1.2378
PPU05296-RA	PpSPH8	1.8924	/	/	-2.5043
PPU05943-RA	PpSPH12	/	/	-2.0461	-2.0644
PPU05952-RA	PpSPH13	/	/	-1.3241	-2.4486
PPU05996-RA	PpSPH14	2.7508	1.7236	/	/
PPU05999-RA	PpSPH15	/	/	/	/
PPU06219-RA	PpSPH16	/	/	-4.6088	-5.5157
PPU06221-RA	PpSPH17	/	/	-1.4943	/
PPU07663-RA	PpSPH19	2.8169	2.4113	1.6087	/
PPU08347-RA	PpSPH21	1.4337	1.0394	1.2433	1.4317
PPU12256-RA	PpSPH26	/	/	-1.6808	-1.3908
PPU12264-RA	PpSPH28	/	-3.4594	/	-8.7814
Antioxidant-related genes					
Superoxide dismutase					

PPU08376-RA	PpSOD1a	/	/	/	-1.0751
PPU08377-RA	PpSOD3	/	/	-1.629	-1.8657
Catalase					
PPU04710-RA	PpCatalase	/	/	-1.358	-1.3316
Peroxidases					
PPU02541-RA	PpHPX1	/	/	/	-1.1128
PPU06118-RA	PpHPX3	/	/	-1.7178	-1.4978
PPU11186-RA	PpHPX4	-1.8005	-1.3424	-2.1403	-1.8014
PPU14440-RA	PpHPX5	/	/	/	-1.0739
PPU15762-RA	PpHPX8	/	/	-3.1492	-3.3186
PPU15763-RA	PpHPX9	/	/	-2.5633	-2.4656
**Genes involved in cuticle formation**					
Cuticle protein					
PPU13753-RA	Apidermin	-1.2429	-1.5673	-2.8051	-2.5436
PPU13755-RA	Apidermin	-1.4803	-2.0923	-2.5348	-2.4279
PPU13756-RA	Apidermin	-2.585	-2.1137	-3.1375	-2.344
PPU03978-RA	CPAP3	-1.7485	-1.4614	-2.3348	-2.7922
PPU10493-RA	CPAP3	/	-1.3825	-2.2484	-2.5618
PPU10496-RA	CPAP3	-1.0887	-1.384	-1.8451	-2.1994
PPU10499-RA	CPAP3	-1.3419	/	/	-1.3966
PPU02385-RA	CPR-RR1	-1.0969	-1.4983	-1.8255	-2.0502
PPU04511-RA	CPR-RR1	/	/	-1.585	-2.3433
PPU06319-RA	CPR-RR1	-1.6208	-1.5025	-3.7182	-4.5295
PPU06320-RA	CPR-RR1	-1.4904	-1.6953	-2.4496	-3.057
PPU06321-RA	CPR-RR1	/	-1.6302	-1.896	-2.6137
PPU06324-RA	CPR-RR1	-1.0473	-1.3394	-2.2165	-2.9442

PPU07134-RA	CPR-RR1	-1.6971	-1.1869	-3.9341	/
PPU10156-RA	CPR-RR1	-1.3785	-3.9318	-4.7549	-5.4553
PPU16556-RA	CPR-RR1	-1.2722	-1.5369	-2.9082	-2.8527
PPU03748-RA	CPR-RR2	/	/	-1.7946	-1.1919
PPU03753-RA	CPR-RR2	/	/	-2.8074	-3.9069
PPU03957-RA	CPR-RR2	-1.2442	/	/	/
PPU07079-RA	CPR-RR-UC	/	-1.4452	-1.6399	-1.4452
PPU07124-RA	CPR-RR-UC	-2.0191		-1.8871	-2.3257
PPU16832-RA	TWEEDLE	-1.9023	-1.6081	-1.8517	-3.0467
Chitnase					
PPU00035-RA	Probable chitinase 3	/	/	/	-1.3098
PPU00925-RA	Probable chitinase 3	6.2164	7.3379	7.611	8.0703
PPU00926-RA	Probable chitinase 3	2.5837	2.7215	3.1804	3.8801
PPU08049-RA	Probable chitinase 3	/	/	-1.3243	-1.5038
PPU07620-RA	Acidic mammalian chitinase	4.521	4.9437	5.4271	5.3851
PPU06384-RA	Chitinase-3-like protein 1	-1.5276	-1.0859	-1.7513	-1.4536
PPU13949-RA	Endochitinase	/	1.5291	1.1058	/
**Genes involved in detoxicated mechanism**					
Glutathione S-transferases					
PPU02083-RA	PpGSTs5	/	/	/	-1.084
PPU02084-RA	PpGSTs6	-1.5868	-1.9843	-2.7554	-2.8992
PPU08124-RA	PpGSTd1	/	-1.0313	-1.1989	-1.1796
PPU09955-RA	PpGSTt2	/	/	-1.1785	-1.2801
PPU13523-RA	PpGSTm1	/	/	-1.2828	-1.3183
PPU14078-RA	PpGSTs4	/	/	-1.0575	-1.1422
PPU16784-RA	PpGSTs1	-1.3079	-1.518	-2.2367	-2.5303

Cytochrome P450					
PPU13898-RA	Cytochrome P450 18a1	/	/	-2.4319	-1.5155
PPU04759-RA	Cytochrome P450 307a1	-1.858	/	-2.4063	-2.1083
PPU05468-RA	Cytochrome P450 315a1, mitochondrial	/	/	-1.417	-1.1822
PPU01686-RA	Cytochrome P450 4C1	/	/	-1.2025	-1.2976
PPU01689-RA	Cytochrome P450 4C1	-2.3209	-1.9007	-3.3003	-3.2014
PPU04105-RA	Cytochrome P450 4C1	/	/	-1.2681	-1.3696
PPU05152-RA	Cytochrome P450 4C1	/	/	-1.6763	-1.5643
PPU09202-RA	Cytochrome P450 4C1	/	/	-2.3444	-3.0923
PPU10700-RA	Cytochrome P450 4C1	-1.4078	/	-2.6439	-1.5621
PPU11441-RA	Cytochrome P450 4C1	/	/	-1.575	-1.842
PPU11488-RA	Cytochrome P450 4C1	/	-1.0787	-1.8132	-1.8883
PPU11489-RA	Cytochrome P450 4C1	/	/	-1.3333	-1.4151
PPU12259-RA	Cytochrome P450 4C1	/	/	-2.087	-2.4451
PPU12506-RA	Cytochrome P450 4C1	/	/	-1.0456	-1.0721
PPU16615-RA	Cytochrome P450 4C1	-1.5025	-1.2864	-3.0494	-3.5828
PPU07154-RA	Cytochrome P450 4c3	/	/	-1.4965	-1.3883
PPU11389-RA	Cytochrome P450 4d2	/	-1.0851	-2.0395	-1.0492
PPU06330-RA	Cytochrome P450 4g15	-1.3129	-1.5097	-3.1771	-3.4844
PPU11624-RA	Cytochrome P450 4g15	/	/	-1.5459	-1.7971
PPU08254-RA	Cytochrome P450 6a2	/	/	/	-1.4909
PPU13608-RA	Cytochrome P450 6a2	/	/	-1.4605	-2.3606
PPU09667-RA	Cytochrome P450 6B1	2.6328	1.6691	1.053	/
PPU16333-RA	Cytochrome P450 6B1	/	/	-2.4275	-1.702
PPU05230-RA	Cytochrome P450 6k1	/	/	-1.0212	-1.083
PPU05637-RA	Cytochrome P450 6k1	1.4961	1.8498	1.7911	2.0858

PPU06135-RA	Cytochrome P450 6k1	/	-1.2029	-2.1325	-2.6353
PPU06204-RA	Cytochrome P450 6k1	/	-1.0218	-1.5583	-1.7062
PPU08018-RA	Cytochrome P450 6k1	/	/	-1.5614	-1.9195
PPU09671-RA	Cytochrome P450 6k1	/	/	/	-1.4689
PPU10814-RA	Cytochrome P450 6k1	/	/	-1.5804	-1.4719
PPU13179-RA	Cytochrome P450 6k1	/	/	-1.2969	-1.1652
PPU13443-RA	Cytochrome P450 6l1	/	/	-1.5103	-2.1572
PPU01260-RA	Cytochrome P450 9e2	/	/	-1.4089	-1.5822
PPU01268-RA	Cytochrome P450 9e2	/	-4.5236	-3.6167	-4.5236
PPU04550-RA	Cytochrome P450 9e2	/	/	/	-1.167
PPU04551-RA	Cytochrome P450 9e2	/	-1.35	-1.6129	-2.1435
PPU05935-RA	Cytochrome P450 9e2	/	/	-1.1104	-1.1272
PPU05940-RA	Cytochrome P450 9e2	/	/	-1.1174	-1.7015
PPU05941-RA	Cytochrome P450 9e2	/	/	/	-1.3307
PPU06977-RA	Cytochrome P450 9e2	/	/	/	-1.2548
PPU10857-RA	Cytochrome P450 9e2	/	/	-1.099	-1.4273
PPU12288-RA	Cytochrome P450 9e2	/	/	-1.0133	-1.2579
PPU12662-RA	Cytochrome P450 9e2	/	-1.0133	-1.8694	-2.442
PPU13444-RA	Cytochrome P450 9e2	/	-1.6272	-2.4525	-1.656
PPU03759-RA	Cytochrome P450 CYP12A2	/	/	-1.2511	-1.1748
PPU06326-RA	Probable cytochrome P450 305a1	/	/	/	-1.9254
PPU15585-RA	Probable cytochrome P450 305a1	/	/	-1.1137	-1.0241
PPU11388-RA	Probable cytochrome P450 4p2	/	-1.017	-1.3024	/
PPU02279-RA	Probable cytochrome P450 6a14	/	/	-1.2434	-1.1593
PPU03219-RA	Probable cytochrome P450 6a14	/	-1.2575	/	/
PPU03487-RA	Probable cytochrome P450 6a14	/	-1.2164	-1.7784	-2.1374

PPU07306-RA	Probable cytochrome P450 6a14	/	-1.1119	-1.8158	-2.1086
PPU08103-RA	Probable cytochrome P450 6a14	/	-1.3642	-2.5263	-2.7656
PPU09664-RA	Probable cytochrome P450 6a14	/	-1.0141	-1.554	-1.9528
PPU09666-RA	Probable cytochrome P450 6a14	/	/	-1.2949	-1.5516
PPU14016-RA	Probable cytochrome P450 6a14	/	-1.925	-2.2139	-3.0859
PPU15214-RA	Probable cytochrome P450 6a14	/	/	-1.3318	-1.2814
PPU15229-RA	Probable cytochrome P450 6a14	/	-1.3249	-2.1707	-2.0003
PPU14017-RA	Probable cytochrome P450 6a20	-1.715	-1.5522	-1.7895	-1.5767
PPU07305-RA	Probable cytochrome P450 6a23	/	/	-1.3041	-1.4512
PPU05229-RA	Probable cytochrome P450 6g2	/	/	-1.0974	-1.4025
ATP-binding cassette transporter					
PPU11283-RA	Multidrug resistance-associated protein 4	2.907	3.02	2.8881	2.5382
PPU11747-RA	Probable multidrug resistance-associated protein lethal(2)03659	1.2625	/	/	/
PPU10962-RA	Multidrug resistance-associated protein 4	1.1988	/	/	/
PPU10959-RA	Probable multidrug resistance-associated protein lethal(2)03659	1.0893	1.0522	1.3083	1.221
PPU03221-RA	Multidrug resistance-associated protein 4	-1.0349	-1.1867	-1.2665	-1.143
PPU05216-RA	ABC transporter G family member 22	-1.0557	/	/	/
PPU03351-RA	ATP-binding cassette sub-family G member 1	-1.1604	-1.512	-2.2272	-2.0021
PPU14473-RA	ATP-binding cassette sub-family G member 1	-1.2093	/	-1.5569	-1.293
PPU03526-RA	Probable multidrug resistance-associated protein lethal(2)03659	/	-1.1214	-1.496	-1.5965
PPU11885-RA	Protein white	/	-1.1988	-2.0087	-2.3613
PPU10117-RA	Multidrug resistance-associated protein 4	/	/	-1.0717	-1.2235

PPU09255-RA	Probable multidrug resistance-associated protein lethal(2)03659	/	/	-1.1712	-1.1289
PPU06865-RA	Probable multidrug resistance-associated protein lethal(2)03659	/	/	-1.2963	-1.5137
PPU10304-RA	Multidrug resistance protein 1	/	/	-1.3243	-1.4894
PPU03350-RA	ATP-binding cassette sub-family G member 4	/	/	-1.7415	-1.9016
PPU12227-RA	Protein brown	/	/	-1.7465	-1.3417
PPU13041-RA	ATP-binding cassette sub-family G member 1	/	/	/	-1.0883
PPU11411-RA	ATP-binding cassette sub-family G member 1	/	/	/	-1.0976
PPU05436-RA	ATP-binding cassette sub-family A member 5	-1.3699	/	/	/
PPU04515-RA	ATP-binding cassette sub-family A member 3	1.4389	1.1568	/	/
Carboxylesterase					
PPU02410-RA	Venom carboxylesterase-6	/	-1.0234	-1.4977	-1.3839
PPU02411-RA	Venom carboxylesterase-6	/	-1.1706	-2.3218	-2.4354
PPU04231-RA	Venom carboxylesterase-6	/	/	/	3.1317
PPU05037-RA	Venom carboxylesterase-6	/	-1.4854	-2.1349	/
PPU09122-RA	Venom carboxylesterase-6	-1.1848	-1.2453	-2.7398	-2.7386
PPU16639-RA	Venom carboxylesterase-6	/	/	-1.766	-1.9111
**Genes involved in olfactory mechanism**					
Odorant binding protein					
PPU00388-RA	Pheromone/general odorant binding protein	/	/	-1.5064	-1.6902
PPU02799-RA	Pheromone/general odorant binding protein	/	/	-1.0513	-1.1229
PPU07034-RA	Pheromone/general odorant binding protein	/	/	-1.4484	-1.496
PPU07039-RA	Pheromone/general odorant binding protein	/	-1.4318	-1.763	-1.7771
PPU07040-RA	Pheromone/general odorant binding protein	/	-1.0394	-1.5313	-1.723
PPU07160-RA	Pheromone/general odorant binding protein	/	/	-3.0744	-2.0028

PPU10873-RA	Pheromone/general odorant binding protein	/	/	-1.7486	-1.5787
PPU10950-RA	Pheromone/general odorant binding protein	/	/	-1.0327	-1.1905
PPU10951-RA	Pheromone/general odorant binding protein	/	/	1.0555	1.0017
PPU10954-RA	Pheromone/general odorant binding protein	/	-1.5565	-1.4022	-1.5794
PPU11325-RA	Pheromone/general odorant binding protein	/	/	-1.0092	-1.0142
PPU13990-RA	Pheromone/general odorant binding protein	/	1.2974	/	/
PPU13991-RA	Pheromone/general odorant binding protein	-2.4144	-2.589	-4.7408	-5.1477
PPU13992-RA	Pheromone/general odorant binding protein	-2.1671	-2.3385	-3.3554	-3.6237
PPU15660-RA	Pheromone/general odorant binding protein	-5.2738	-4.1807	-5.8588	-6.8588
PPU15673-RA	Pheromone/general odorant binding protein	-1.1112	-1.1202	-2.489	-2.761
PPU15674-RA	Pheromone/general odorant binding protein	/	/	-1.7323	-1.6714
PPU15675-RA	Pheromone/general odorant binding protein	/	/	-1.7621	-1.8252
PPU15676-RA	Pheromone/general odorant binding protein	-3.0978	-3.5225	-4.914	-5.1878
PPU15677-RA	Pheromone/general odorant binding protein	-1.2154	-1.2041	-3.2229	-2.7053
PPU15678-RA	Pheromone/general odorant binding protein	-1.5456	/	-2.5392	-2.5136
PPU15680-RA	Pheromone/general odorant binding protein	/	/	-1.9181	-1.8293
PPU15682-RA	Pheromone/general odorant binding protein	-1.5169	-2.1721	/	/
PPU15684-RA	Pheromone/general odorant binding protein	/	/	-2.3587	-2.2913
PPU15685-RA	Pheromone/general odorant binding protein	/	/	-1.2992	-1.2652
PPU15687-RA	Pheromone/general odorant binding protein	/	/	-1.6603	-1.6944
PPU15699-RA	Pheromone/general odorant binding protein	/	/	-1.7509	-1.7038
PPU15701-RA	Pheromone/general odorant binding protein	/	/	-2.2078	-1.7023
PPU04648-RA	Insect odorant-binding protein A10	/	/	-2.6466	-2.8624
PPU04649-RA	Insect odorant-binding protein A10	-1.5674	-1.9955	-2.2165	-2.7486
PPU04651-RA	Insect odorant-binding protein A10	-1.3453	-1.5024	-2.5477	-2.5122
PPU04656-RA	Insect odorant-binding protein A10	/	-1.0529	-1.8243	-2.0475

PPU04657-RA	Insect odorant-binding protein A10	/	-1.0086	-2.1575	-2.1854
Ionotropic receptor					
PPU02488-RA	Ionotropic glutamate receptor	-1.6394	/	-1.6681	/
PPU04653-RA	Ionotropic glutamate receptor	/	/	-1.314	-1.2557
PPU06894-RA	Ionotropic glutamate receptor	/	/	4.1115	/
PPU11721-RA	Ionotropic glutamate receptor	-1.5298	/	/	/
PPU12640-RA	Ionotropic glutamate receptor	-1.7745	-1.4933	-1.8049	-1.788
PPU12641-RA	Ionotropic glutamate receptor	-1.2522	-1.2366	-2.0148	-1.4061
PPU13751-RA	Ionotropic glutamate receptor	/	/	-1.2248	/
PPU13757-RA	Ionotropic glutamate receptor	/	/	-1.1604	/
PPU14661-RA	Ionotropic glutamate receptor	/	/	-1.1651	/
PPU15454-RA	Ionotropic glutamate receptor	/	/	-1.1497	-1.1802
PPU15458-RA	Ionotropic glutamate receptor	/	/	-1.1316	/
Olfactory receptor					
PPU01104-RA	Olfactory receptor, Drosophila	-1.1623	/	/	-1.4461
PPU01105-RA	Olfactory receptor, Drosophila	-1.2361	-1.138	-1.5315	-1.394
PPU01106-RA	Olfactory receptor, Drosophila	/	-1.4656	-1.2561	-1.2512
PPU01108-RA	Olfactory receptor, Drosophila	/	/	-1.7203	/
PPU03580-RA	Olfactory receptor, Drosophila	/	/	/	-3.4507
PPU06205-RA	Olfactory receptor, Drosophila	/	/	/	-1.3399
PPU06948-RA	Olfactory receptor, Drosophila	-1.0147	/	/	/
PPU07371-RA	Olfactory receptor, Drosophila	2.5162	2.2067	/	2.1206
PPU07541-RA	Olfactory receptor, Drosophila	-1.6189	-1.4843	-1.6107	-1.4619
PPU07721-RA	Olfactory receptor, Drosophila	-1.0085	/	/	/
PPU08352-RA	Olfactory receptor, Drosophila	/	/	-4.2695	/
PPU09317-RA	Olfactory receptor, Drosophila	/	-1.8097	/	-2.3547

PPU09328-RA	Olfactory receptor, Drosophila	-1.0863	-1.0311	-1.5187	/
PPU09329-RA	Olfactory receptor, Drosophila	/	/	-1.0233	/
PPU09997-RA	Olfactory receptor, Drosophila	/	/	-1.6843	/
PPU11832-RA	Olfactory receptor, Drosophila	/	/	-2.8365	/
PPU12457-RA	Olfactory receptor, Drosophila	-1.122	-1.1929	-1.6468	-1.7193
PPU12654-RA	Olfactory receptor, Drosophila	/	/	-1.2232	-1.3837
PPU14379-RA	Olfactory receptor, Drosophila	/	/	-1.5455	-1.4531

Table S6. Primers used for rt-qPCR on gene expression.

Gene ID		Sequence (5'-3')	Size(bp)
PPU04036-RA	-F-	ACGAGAAGAGCATTGGCATT	143
	-R-	TGTGGCCAATCAACGAATAA	
PPU16903-RA	-F-	ACGGAGTAGAAGCTGGCAAA	122
	-R-	AGGGTGTTTGAGACCAGTGC	
PPU04173-RA	-F-	GTGCGAAGATTTGTGCAAGA	154
	-R-	AGCTCGAGTCGTCGGAATAA	
PPU09907-RA	-F-	CGCGAGATCTTTTGAGAGGC	135
	-R-	TGAGGTTGTTGTTGCTGAGC	
PPU14446-RA	-F-	CCAACAGTTACACAGCACCC	107
	-R-	GTTGTTGATGTCCGTGTGCT	
PPU03121-RA	-F-	CTCGTCAGCGTTACCAAACA	113
	-R-	TGAACAGCTTTGCGTTTGAC	
PPU06179-RA	-F-	AAAGACTTCGCCGACTACGA	147
	-R-	GATCCGTGATTCGTGTGTTG	
PPU12674-RA	-F-	TGAAGCTCCTACTCGTTGTTG	124
	-R-	TCCTCGTCGACTACTGCTTC	
PPU12920-RA	-F-	ACCCTTACACCGGACATCTG	99
	-R-	TCTCAGAAACGCATCGCAAG	
PPU04479-RA	-F-	CGACGTCTTAGAACAGCAGC	91
	-R-	CTCCATCAAAGAGTGCGTCG	
PPU05991-RA	-F-	GTTCAACTTCGACGACACCC	112
	-R-	CGAACCCTTGCCAGTGTTAC	
PPU08376-RA	-F-	GCGGTCTTAACAAGGATGGA	139
	-R-	ACGTGGCGGACTGTATCTTC	
PPU16556-RA	-F-	TGACGAAAACGGCTTCCAAG	119
	-R-	TGTAGTCGTTGTCCTCCTCG	
PPU00926-RA	-F-	CTTAGCCAAGCCCAAGTGTC	95
	-R-	CTCCCGAGCACTGGTAGTAG	
PPU07620-RA	-F-	ATCCTGGACAAATGGGCTGA	146
	-R-	ACCTTCGAGTACTTTGCCGA	
PPU04515-RA	-F-	GGACACGTTCGCCAAGTATC	137
	-R-	ACATCTGTCGGCGAGAGTAG	
PPU05423-RA	-F-	ACTTCGTTTTCCTGCTCGTC	101
	-R-	TCCCTTCATGATGCAGTCGA	
PPU13684-RA	-F-	GTCGACCAGAAATCTCGTGC	138
	-R-	GTTGGCCTTGAGAGTCTCCT	
PPU03355-RA	-F-	CGGCACAAACAGAGTACGAG	165
	-R-	GCACCTACATCTCCTCCGAA	
PPU04073-RA	-F-	GCTGCTACTGTTGGATCTGC	90
	-R-	AGCCAAGTGATGAGTGAGCT	

PPU05385-RA	-F-	CTATGTTCCCGAAAGCCACG	138
	-R-	CGTAGTGACCAATGGCGAAG	
PPU16911-RA	-F-	TCGTTGGCTGCGTTGTTTTA	133
	-R-	CCGTAAGCCCAGTCGTATGA	
PPU07620-RA	-F-	ATCCTGGACAAATGGGCTGA	146
	-R-	ACCTTCGAGTACTTTGCCGA	
PPU07050-RA	-F-	TGATCATCAAGCAAGCTGGC	112
	-R-	AATTTGGGCATTGTGTCGCT	
18s rRNA	-F-	CGAGCGATGAACCGACAG	
	-R-	CGGGGAGGTAGTGACGAA	
